# Targeting Antigen‐Presenting Cells to Enhance the Tumor‐Spleen Immunity Cycle through Liposome‐Neoantigen Vaccine

**DOI:** 10.1002/advs.202500021

**Published:** 2025-03-24

**Authors:** Yu Xu, Bing Wang, Yue Huang, JianPing Liao, Chenyi Wu, Chenxi Zhou, Zishi Kang, Shiyang Jiang, Bing‐Chen Wu, Da Zhang, Ruihua Xu, Xiaolong Liu, Feng Wang

**Affiliations:** ^1^ Department of Medical Oncology Sun Yat‐sen University Cancer Center State Key Laboratory of Oncology in South China Collaborative Innovation Center for Cancer Medicine Sun Yat‐sen University Guangzhou 510060 P. R. China; ^2^ Research Unit of Precision Diagnosis and Treatment for Gastrointestinal Cancer Chinese Academy of Medical Sciences Guangzhou 510060 P. R. China; ^3^ Sun Yat‐sen University Cancer Center State Key Laboratory of Oncology in South China Collaborative Innovation Center for Cancer Medicine Sun Yat‐sen University Guangzhou 510060 P. R. China; ^4^ Department of Medical Oncology Sun Yat‐sen University Cancer Center State Key Laboratory of Oncology in South China Guangdong Provincial Clinical Research Center for Cancer Sun Yat‐sen University Guangzhou 510060 P. R. China; ^5^ The United Innovation of Mengchao Hepatobiliary Technology Key Laboratory of Fujian Province Mengchao Hepatobiliary Hospital of Fujian Medical University Fuzhou 350025 P. R. China; ^6^ CAS Key Laboratory of Design and Assembly of Functional Nanostructures Fujian Institute of Research on the Structure of Matter Chinese Academy of Sciences Fuzhou 350002 P. R. China; ^7^ Mengchao Med‐X Center Fuzhou University Fuzhou 350116 P. R. China; ^8^ Fujian Agriculture and Forestry University Fuzhou 350002 P. R. China

**Keywords:** neoantigen vaccine, spleen‐tumor immunity, targeting antigen‐presenting cells, tumor microenvironment, tumor‐associated macrophages

## Abstract

Effective immune responses in both the spleen and the tumor microenvironment are crucial for cancer immunotherapy. However, delivery of neoantigen peptide vaccines to antigen‐presenting cells (APCs) at these sites remains challenging. In this study, LNPs^D18^, a cationic liposomal formulation that targets and enhances APC uptake at both sites without modifying the targeting ligands is developed. By co‐delivering tumor‐specific neoantigens and a cholesterol‐coupled toll‐like receptor 9 (TLR9) agonist within LNP‐vax^D18^, an approximately 60‐fold increase in dendritic cell uptake compared to neoantigen‐adjuvant mixtures is achieved. Intravenous administration of the liposome‐neoantigen peptide vaccine targets both the spleen and the tumor, boosting splenic DC activation, increasing M1‐type tumor‐associated macrophages, and elevating tumor cytokine levels. This reshapes the tumor microenvironment, enhancing IFN‐γ‐producing CD8^+^ T cells and TCF1^+^CD8^+^ T cells within tumors. These outcomes significantly inhibit established tumor growth compared to nontargeted lipid‐based nanovaccine formulations, resulting in improved survival in orthotopic hepatocellular carcinoma and colorectal cancer models. The findings highlight the importance of targeting APCs in both the spleen and tumors to optimize the therapeutic efficacy of liposome‐neoantigen vaccines in cancer treatment.

## Graphical abstract

LNPs^D18^, a cationic liposome that enhances antigen‐presenting cell uptake in the spleen and tumor without targeting ligands is developed. Co‐delivering neoantigens and a TLR9 agonist significantly improve DC activation, tumor immunity, and survival in liver and colorectal cancer models, demonstrating the importance of dual‐site APC targeting for cancer immunotherapy.

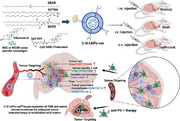

## 1 Introduction

Recent advancements in rapid genomic sequencing have paved the way for personalized cancer vaccines (PCVs) that target tumor‐specific neoantigen peptides.^[^ ^1^, ^2^, ^3^ ^]^ These highly immunogenic, tumor‐specific, and easily fabricated neoantigen peptides have minimal side effects and hold great promise as PCVs for post‐surgical tumor suppression in melanoma,^[^ ^4^ ^]^ pancreatic cancer,^[^ ^5^ ^]^ hepatocellular carcinoma (HCC),^[^ ^6^ ^]^ glioblastoma,^[^ ^7^ ^]^ and lung cancer.^[^ ^8^ ^]^ Generating intratumoral T‐cell responses specific to neoantigens is crucial for targeted tumor elimination and improved survival rates.^[^ ^9^, ^10^ ^]^ This process relies heavily on the activation of antigen‐presenting cells (APCs) in the spleen or lymph nodes, which prime cytotoxic T cells to effectively eradicate cancer cells.^[^ ^11^, ^12^, ^13^, ^14^ ^]^ Additionally, CD205 expression on APCs plays a crucial role in endocytosis and targeted APCs delivery to PCVs. This interaction triggers innate immune sensors such as Toll‐like receptors (TLRs), and NOD‐like receptors, to induce cytokine production (IL‐12, TNF‐α, and IFN‐α).^[^ ^15^ ^]^ This in turn allows APCs to present antigens, leading to robust antitumor T‐cell responses.^[^ ^16^ ^]^ However, most neoantigen peptide vaccines in clinical trials rely on passive encounters with APCs^[^ ^17^, ^18^, ^19^, ^20^ ^]^ and lack targeted delivery mechanisms. This limitation results in insufficient antigen uptake, weak CD8^+^ T‐cell response, and reduced vaccine efficacy. Moreover, tumor‐associated macrophages (TAMs) suppress CD8^+^ T‐cell mediated immunity by fostering an immunosuppressive tumor microenvironment (TME) that shields tumor cells from immune attack.^[^ ^21^ ^]^ As a result, the response rates of patients to neoantigen peptide vaccines are comparatively low, leading to the unsuccessful eradication of solid tumors at advanced stages.

TAMs are present in nearly all solid tumors.^[^ ^22^ ^]^ TAMs exhibit characteristics similar to those of their Th1 counterparts (M1), which have proinflammatory and tumor‐suppressive properties. M1‐type TAMs induce tumor rejection in various tumor models, thereby boosting T‐cell based immunotherapy.^[^ ^23^, ^24^ ^]^ By promoting a favorable tumor immune environment, M1‐like TAMs facilitate the activation and proliferation of cytotoxic T cells, thereby improving the overall efficacy of cancer treatment. The spleen serves as a reservoir for monocytes, with many of these monocytes migrating to tumors as TAM precursors. Notably, splenic ablation has been associated with reduced TAM responses and delayed tumor growth.^[^ ^25^ ^]^ In addition, CpG‐ODN can prompt monocytic myeloid‐derived suppressor cells (MDSCs) to differentiate into M1‐type macrophages and promote tumor elimination.^[^ ^26^ ^]^ As a Toll‐like receptor 9 (TLR9) agonist, CpG‐ODN can stimulate the maturation and cytokine production of dendritic cells (DCs), to enhance the antitumor immune response. More importantly, PCVs combined with CpG‐ODN‐conjugated nanoparticles significantly boost DC activation and CD8^+^ T‐cell cross‐presentation, requiring lower doses and causing less toxicity than PCVs alone.^[^ ^27^, ^28^, ^29^ ^]^ Among the different types of nanoparticles, liposome/lipid nanoparticles (LNPs) offer a range of advantages over inorganic nanoparticles, including versatile cargo transport, optimized loading, biocompatibility, low toxicity, surface modification capabilities, and controlled release properties.^[^ ^30^, ^31^, ^32^ ^]^ Several cancer neoantigen vaccines utilizing the LNP technology are currently undergoing clinical trials for the treatment of melanoma, triple‐negative breast cancer, gastrointestinal cancer, and HPV‐positive cancer (NCT03897881, NCT02316457, NCT03480152, and NCT03418480). Furthermore, the use of spleen‐ or splenic DC‐targeting LNPs for cancer vaccine delivery is a powerful technology for the fight against cancer.^[^ ^33^, ^34^, ^35^ ^]^ However, most studies have focused on ligand modifications to target DC receptors, such as Clec9a, XCR1, Clec12A, Clec4A4, and mannose,^[^ ^36^ ^]^ and LNPs with inherent DC‐targeting capabilities remain limited.^[^ ^33^ ^]^ Therefore, simultaneously targeting splenic DC activation and increasing the population of M1‐like TAMs using LNPs could represent a promising strategy to further enhance the effectiveness of neoantigen peptide vaccines in activating T cells against tumors.

**Figure 1:**
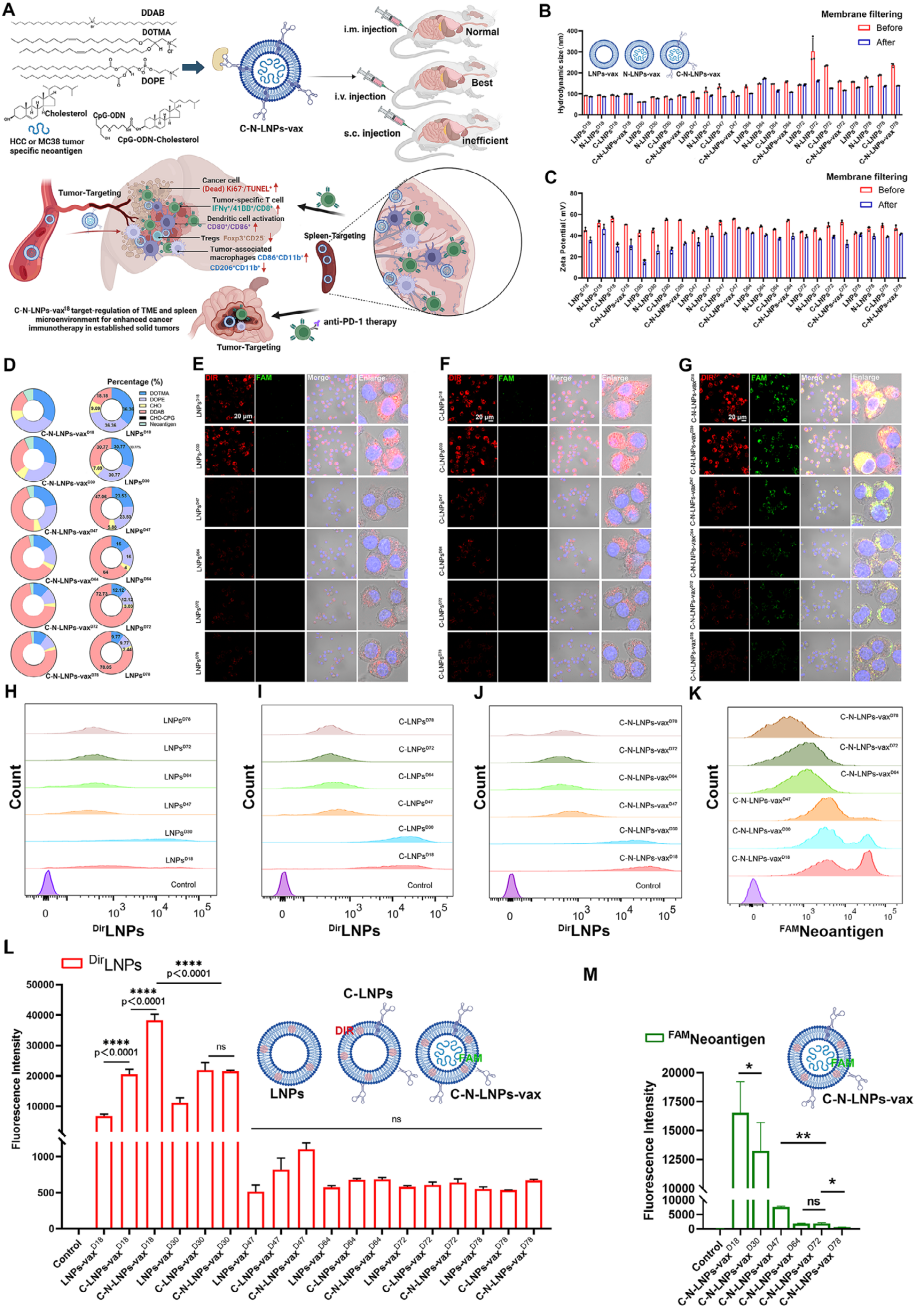
A) Schematic illustration of the preparation of the LNPs‐neoantigen vaccine (C‐N‐LNPs‐vax) to target the regulation of tumors and spleen antigen‐presenting cells for enhanced antitumor immunity. Illustration created by BioRender.com. B) Hydrodynamic diameter and C) the zeta potential of different formulations of LNPs, neoantigen alone loaded LNPs (N‐LNPs‐vax), CHO‐CpG alone loaded LNPs (C‐LNPs), or C‐N‐LNPs‐vax before and after extrusion through the filtration by a membrane (0.25 µm), *n* = 3. D) Different percentages of DOTMA, DDAB, CHO, and DOPE in LNP formulations. E–K) Quantification of Dir or FAM fluorescence intensity from DCs2.4 cells after co‐incubation of Dir‐labeled LNPs, CHO‐CpG loaded Dir‐labeled LNPs (C‐^Dir^LNPs‐vax), or ^FAM^CHO‐CpG loaded Dir‐labeled LNPs (C‐^FAM^N‐^Dir^LNPs‐vax) with different formulations for 12 h and analyzed by confocal microscopy (CLSM) and flow cytometry (FCM), respectively. L) Representative quantification of the mean fluorescence intensity of Dir from LNPs, C‐^Dir^LNPs‐vax, or C‐N‐^Dir^LNPs‐vax by FCM analysis (*n* = 3). M) Representative quantification of the mean fluorescence intensity of FAM from C‐^FAM^N‐LNPs‐vax with different formulations by FCM analysis (*n* = 3). Statistical analysis was performed with ANOVA analysis, ^*^p < 0.05, ^**^p < 0.01. Data are expressed as mean ± SD.

Herein, we screened various cationic liposome (LNP) formulations and identified an LNP^D18^, that is superior for targeting APCs in both the spleen and tumors without the need for modification of targeting ligands (**Figure** **1**A). By incorporating cholesterol (CHO) linked and thio‐modified CpG‐ODNs and tumor‐specific neoantigen peptides, the LNP^D18^‐based neoantigen vaccine significantly enhanced DCs maturation in vitro, surpassing the effects of a simple mixture of adjuvant and neoantigen peptides. In therapeutic tumor models, our LNP^D18^‐based nanovaccines facilitated preferential delivery, activated splenic DCs, and increased the population of M1‐TAMs in tumors through dual targeting of the spleen and tumors. These advantages translate into elevated levels of IFN‐γ‐producing CD8^+^ T cells and TCF1^+^CD8^+^ T cells in tumors, leading to suppression of tumor growth compared to the standard neoantigen‐adjuvant mixture and commercial vaccine formulation such as SM102 and MC3 in the Hepa1‐6 cell‐based orthotopic HCC models and MC38 cell‐based colorectal cancer models by prolonging survival in mice. Combining our LNP^D18^‐based vaccine with α‐programmed death‐1 (αPD1) monoclonal antibody further enhanced therapeutic outcomes in both tumor models. Our study offers valuable insights into targeting APCs to modulate the spleen‐tumor immunity cycle to eradicate solid tumors.

## 2 Results

### 2.1 Screening Liposome and Liposome‐Based Vaccines for Efficient Uptake by APCs

Typical LNPs comprise four components: cationic lipids, polyethylene glycol (PEG) lipids, cholesterol (CHO), and phospholipid.^[^ ^37^, ^38^ ^]^ However, PEGylated drugs induce anti‐PEG antibody production in animal models and humans, leading to accelerated clearance and compromised therapeutic efficacy.^[^ ^39^ ^]^ Severe immune reactions such as hypersensitivity reactions and fatal outcomes are associated with the presence of anti‐PEG antibodies.^[^ ^40^ ^]^ To address this, we developed PEG‐free LNPs using cationic lipids, such as *N*‐[1‐(2,3‐dioleyloxy)propyl]‐*N*,*N*,*N*‐trimethylammonium chloride (DOTMA) and dimethyl dioctadecyl ammonium (DDAB), along with the helper lipid CHO and zwitterionic lipids (1,2‐dioleoyl‐sn‐glycero‐3‐phosphoethanolamine) (DOPE). By using the ethanol injection method^[^ ^41^ ^]^ and an extrusion step, we generated a series of LNP formulations (LNPs^D18^, LNPs^D30^, LNPs^D47^, LNPs^D64^, LNPs^D72^, and LNPs^D78^) with different molar ratios of DOTMA, DOPE, CHO, and DDAB. We used dynamic light scattering (DLS) to analyze LNP size and zeta potential, and the results showed that the hydrodynamic diameter of the LNPs from 61.30 to 143.5 nm coupled with relatively low PDI value, and the zeta potentials were from +15.57 to +40.93 mV (Figure 1B–D and Figure S1, Supporting Information).

To assess the efficiency of different LNP formulations in cellular uptake by APCs, we tracked LNPs using DiR fluorescence dye. The DiR‐labeled LNP formulations were co‐incubated with DCs2.4 cell lines for 12 h and then imaged by confocal microscopy (CLSM) and flow cytometry (FCM) (Figure 1E,M). The results revealed strong red fluorescence in DCs2.4 cells treated with ^Dir^LNPs^D18^ and ^Dir^LNPs^D30^, whereas the red fluorescence was notably weaker in DCs2.4 cells treated with ^Dir^LNPs^D47^, ^Dir^LNPs^D64^, ^Dir^LNPs^D72^, or ^Dir^LNPs^D78^. In addition, to explore the impact of CHO‐CpG modification on the DC uptake of LNPs, the Dir‐labeled CHO‐CpG‐modified LNPs formulation was further co‐incubated with DCs2.4 cells for 12 h. CLSM imaging revealed enhanced LNP uptake by DCs when CHO‐CpG was incorporated into LNPs^D18^ and LNPs^D30^, likely because CpG‐ODN acted as a CD205 ligand on the DCs.^[^ ^42^ ^]^ Conversely, no significant difference in LNP uptake was observed with or without CHO‐CpG modification of LNPs^D47^, LNPs^D64^, LNPs^D72^, or LNPs^D78^, indicating the limited targeting efficacy of CHO‐CpG in these formulations.

After loading Hepa1‐6 liver tumor cell‐specific neoantigens (WDTCTTYKWQKTLEGHD), the C‐N‐LNPs‐vax formulations were obtained. The hydrodynamic diameters and zeta potentials of these C‐N‐LNPs‐vax formulations further increased because of the adsorption of neoantigen peptides through electrostatic interactions and hydrophobic effects. The UV–Vis spectra of these C‐N‐LNPs‐vax formulations displayed a unique absorption peak at 510 nm, further confirming the successful loading of the neoantigens into the LNPs (Figure S2, Supporting Information). After co‐incubated with DCs2.4 for 12 h, a much higher mean fluorescence intensity (MFI) of FAM was observed in C‐N‐LNPs‐vax^D18^‐treated DCs2.4 cells compared to that in C‐N‐LNPs‐vax^D30^, C‐N‐LNPs‐vax^D47^, C‐N‐LNPs‐vax^D64^, C‐N‐LNPs‐vax^D72^, and C‐N‐LNPs‐vax^D78^‐treated cells through FCM. Based on the neoantigen uptake efficiency of C‐N‐LNPs‐vax, we selected the LNP‐vax^D18^ formulation for further investigation. These results demonstrate that the LNPs^D18^ and LNP^D18^‐based vaccines exhibited superior cellular uptake by APCs.

Biosafety of LNP‐neoantigen vaccines is crucial for their potential clinical use. Initially, we performed the CCK‐8 cell viability test. The results indicated that exposure to C‐N‐LNPs‐vax^D18^ did not show significant toxicity, with cell viability above 102.74% and 92.22% in DCs2.4 after 24 or 48 h of co‐incubation, respectively, compared to the control group (approximately 100%) (Figure S3, Supporting Information). Additionally, we investigated the in vivo biosafety of LNP‐neoantigen vaccines by analyzing serological and physiological biochemical indices in healthy mice after intravenous injection (i.v.) with a therapeutic dose of the vaccines. No significant changes in the levels of alkaline phosphatase (ALP), alanine aminotransferase (ALT), albumin (ALB), or aspartate aminotransferase (AST) were observed after the administration of LNP‐neoantigen vaccines at 14 days compared to the PBS‐treated group (Figure S4, Supporting Information). These findings demonstrated that the C‐N‐LNPs‐vax^D18^ formulation was safe.

### 2.2 Characteristics of C‐N‐LNPs‐vax^D18^ In Vitro and In Vivo

**Figure 2:**
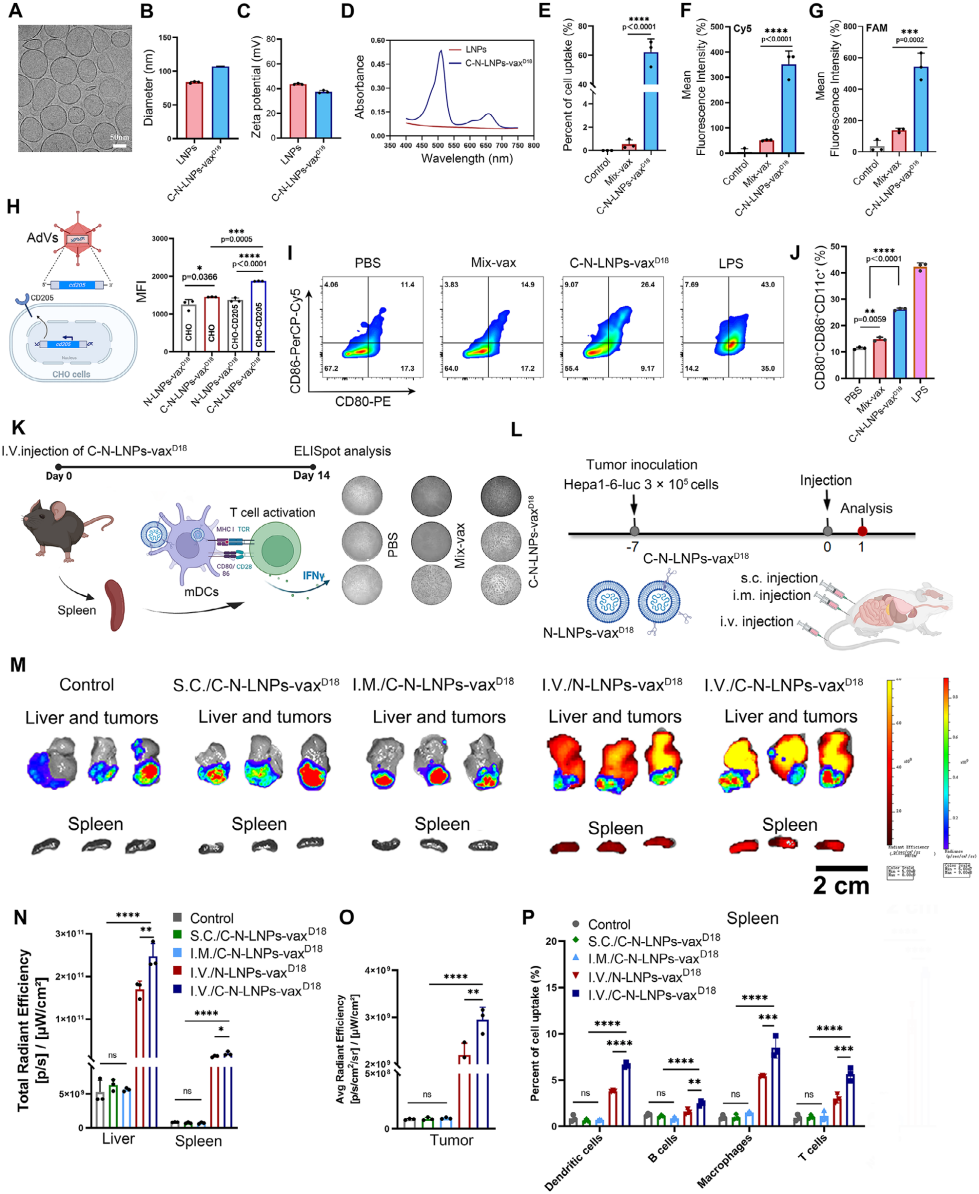
A) Representative electron cryo‐microscopy (cryo‐EM) images of C‐N‐LNPs‐vax^D18^. Scale bar, 50 nm. B) The size distribution and C) surface zeta potential of C‐N‐LNPs‐vax ^D18^ and LNPs was measured by DLS (*n* = 3). The table represents the average size, PDI, and zeta potentials (*n* = 3). D) The absorption of ^Cy5^C‐^FAM^N‐LNPs‐vax^D18^ (blue line) and LNPs (red line) from 400 to 800 nm. The absorption peak of FAM was at 490 nm from neoantigens, and the absorption peak of Cy5 was at 660 nm from CHO‐CpG. E) Quantification of the presence of Cy5 and FAM fluorescence in DCs2.4 cells after being treated with Mix‐vax (CHO‐^Cy5^CpG and ^FAM^neoantigen) and ^Cy5^C‐^FAM^N‐LNPs‐vax^D18^ for 12 h co‐incubation, which were determined by FCM. *N* = 3. Presence of Cy5 fluorescence (F) or FAM fluorescence (G) in DCs2.4 cells. H) Schematic illustration of transient co‐transfection of CD205 gene to CHO cells by adenovirus. I) Cellular uptake of C‐N‐LNPs‐vax^D18^ by CD205^+^CHO cells or CD205^−^CHO cells for 12 h co‐incubation, which was measured by FCM (*n* = 3). J) Percentage of CD80 and CD86 co‐expression in CD11c^+^ BMDCs after co‐incubation of PBS, Mix‐vax, C‐N‐LNPs‐vax^D18^, or lipopolysaccharide (LPS, as positive control), *n* = 3. K) Schematic illustration of ELISPOT assay analysis of IFN‐γ spot‐formation of T cells that isolated from the spleen of female C57BL/6 mice after i.v. injection of PBS, Mix‐vax, or C‐N‐ LNPs‐vax^D18^ for 14 days, and then re‐stimulation of T cells by Hepa1‐6‐specific neoantigens for 48 h. Illustration created by BioRender.com. M) Different immune cell uptake of N‐LNPs‐vax^D18^ (without CHO‐CpG) or C‐N‐LNPs‐vax^D18^ with different administrations, i.v., i.m., or s.c. injection (*n* = 3). N) Fluorescence intensity per gram in liver and spleen and O) tumor from livers (*n* = 3). P) The different immune cell (DCs cells, B cells, macrophages, and T cells) uptake capacity of N‐LNPs‐vax^D18^ or C‐N‐LNPs‐vax^D18^ with different administration routes, i.v., i.m., or s.c. injection (*n* = 3). Statistical analysis was performed with ANOVA analysis, ^*^p < 0.05, ^**^p < 0.01, ^***^p < 0.001, ^****^p < 0.0001. Data are expressed as mean ± SD.

Electron cryomicroscopy (cryo‐EM) imaging revealed a typical liposome structure comprising a bilayer membrane and a hydrophilic core of C‐N‐LNPs‐vax^D18^ (**Figure** **2**A). The hydrodynamic diameter was 134 nm and the zeta potential was 37.4 mV (Figure 2B,C). The UV–Vis spectra showed two characteristic absorption peaks at 510 and 660 nm in C‐N‐LNPs‐vax^D18^ compared to the LNPs alone, indicating effective co‐loading of the neoantigen and CHO‐CpG onto the LNPs (Figure 2D). To evaluate the efficiency of co‐delivery of neoantigens and adjuvants via C‐N‐LNPs‐vax^D18^ to APCs, we co‐incubated DCs.2.4 cells with C‐N‐LNPs‐vax^D18^ for 12 h. A standard mixture of neoantigens and adjuvants (Mix‐vax) was used as a control. As shown in Figure S5 (Supporting Information), DCs.2.4 cells treated with C‐N‐LNPs‐vax^D18^ displayed significantly higher levels of red (CHO‐^Cy5^CPG) and green (^FAM^neoantigen) fluorescence than Mix‐vax‐treated DCs.2.4 cells. FCM analysis demonstrated an approximately 60‐fold increase in the co‐delivery efficiency of neoantigen and CHO‐CpG in C‐N‐LNPs‐vax^D18^‐treated DCs.2.4 cells (cells showing dual positive Cy5 and FAM dye in Q3 quadrant) compared to Mix‐vax‐treated DCs2.4 cells (Figure 2E–G, Supporting Information). The impact of CpG‐CHO on the intracellular uptake was further analyzed using Chinese hamster ovary (CHO) cells expressing mouse CD205, as confirmed by Western blotting (Figure 2H and Figure S6, Supporting Information). A higher increase in C‐N‐LNPs‐vax^D18^ uptake was observed in CHO cells overexpressing CD205 than in cells treated with N‐LNPs‐vax^D18^ without CHO‐CpG and was relatively higher than that of C‐N‐LNPs‐vax^D18^ treated CHO cells without CD205 overexpression *(p* = 0.0005). These results indicated that the CD205 receptor further enhanced the cellular uptake of C‐N‐LNPs‐vax^D18^. To further verify the importance of CD205 in cellular uptake of C‐N‐LNPs‐vax^D18^ by bone marrow‐derived dendritic cells (BMDCs), we blocked the CD205 by anti‐CD205 antibodies, and then co‐incubation with C‐N‐LNPs‐vax^D18^. As shown in Figure S7 (Supporting Information), the uptake efficiency of C‐N‐LNPs‐vax^D18^ by BMDCs was significantly reduced by CD205 blocking. These results suggest that CHO‐CpG not only functions as a TLR9 agonist but may also contribute to the cellular uptake of C‐N‐LNPs‐vax^D18^ by APCs. We further assessed the water stability of C‐N‐LNPs‐vax^D18^ in a solution containing 10% fetal bovine serum (FBS) to mimic blood circulation (Figure S8, Supporting Information). The results demonstrated that C‐N‐LNPs‐vax^D18^ were relatively stable in a 10% FBS solution, with no significant changes in nanoparticle size or PDI over a 10‐day monitoring period. This result indicated that the formulation could retain its stability in serum, which is crucial for its potential in vivo applications.

To examine the activation capacity of C‐N‐LNPs‐vax^D18^ to BMDCs, FCM analysis was performed to assess the activation of CD11c^+^BMDCs by staining for the co‐stimulatory molecules CD80 and CD86 (Figure 2I,L, and Figure S9, Supporting Information). After co‐incubation for 48 h, CD11c^+^BMDCs treated with C‐N‐LNPs‐vax^D18^ showed significant upregulation of CD80 and CD86 co‐expression (26.4%) compared to BMDCs treated with Mix‐vax (14.9%) and PBS (11.4%). To monitor the immune response of mice who received C‐N‐LNPs‐vax^D18^ vaccination, an in vitro IFN‐γ ELISpot assay was performed (Figure 2K). The ELISPOT assay revealed that mice receiving C‐N‐LNPs‐vax^D18^ treatment displayed higher secretion of IFN‐γ compared to those treated with Mix‐vax when compared to the corresponding negative control, indicating the effective activation of IFN‐γ^+^‐producing CD8^+^ T cells (Tc1). These results indicate the efficient activation of APCs and T‐cell responses by C‐N‐LNPs‐vax^D18^.

### 2.3 I.V. Injection of C‐N‐LNPs‐vax^D18^ Induces IFN‐γ‐Producing CD8^+^ T Cells

Preclinical studies have emphasized the potential of tumor‐specific cancer vaccines to modulate the tumor immune microenvironment (TiME) and enhance antitumor effects.^[^ ^43^, ^44^ ^]^ Additionally, our recent studies have suggested that spleen‐targeting cancer vaccines can enhance APC activation, thereby enhancing T‐cell mediated antitumor immunotherapy.^[^ ^34^ ^]^ We hypothesized that concurrently targeting and modulating both the TME and spleen is critical for amplifying tumor‐spleen immunity and augmenting the efficacy of the LNP‐neoantigen vaccine against cancer. However, the route of vaccine administration can also affect the antitumor capacity of neoantigen‐specific CD8^+^ T cells.^[^ ^45^ ^]^ Therefore, we investigated the antitumor efficacy of C‐N‐LNPs‐vax^D18^ by subcutaneous (s.c.), intramuscular (i.m.), and intravenous (i.v.) administration in an orthotopic Hepa1‐6 liver tumor model (Figure 2L). The control group received i.v. injection with PBS. Initially, the successful establishment of tumor models was confirmed using bioluminescence imaging. After 24 h of administration of C‐N‐LNPs‐vax^D18^ via different routes, the livers with tumors and spleens from treated mice were collected and imaged using bioluminescence and fluorescence imaging. As shown in Figure 2M–O, intravenous injection of C‐N‐LNPs‐vax^D18^ resulted in significantly higher Dir fluorescence levels in the spleen, liver, and tumors than intravenous injection of s.c. or i.m. injection of C‐N‐LNPs‐vax^D18^, indicating the efficient targeting ability of C‐N‐LNPs‐vax^D18^ in the spleen and tumor tissues through i.v. injection.

To further verify the delivery of C‐N‐LNPs‐vax^D18^ to splenic APCs, immune cell types, including DCs, B cells, and macrophages, were collected from the spleen and analyzed using FCM with specific biomarker staining (Figure 2P and Figure S10, Supporting Information). We found that C‐N‐LNPs‐vax^D18^ administered via the i.v. injection was more effectively taken up by DCs and macrophages compared to the s.c. or i.m. injection of C‐N‐LNPs‐vax^D18^. The excellent uptake of C‐N‐LNPs‐vax^D18^ into the spleen via macrophages and DCs may play a key role in activating adaptive immune responses. These results highlight the importance of the route of administration in determining immune cell uptake and activation of C‐N‐LNPs‐vax^D18^, which may affect the effectiveness of antitumor immune responses. Based on these results, we hypothesized that tumor suppression by i.v. injection of C‐N‐LNPs‐vax^D18^ should be superior to that by s.c. or i.m. injection of C‐N‐LNPs‐vax^D18^.

**Figure 3:**
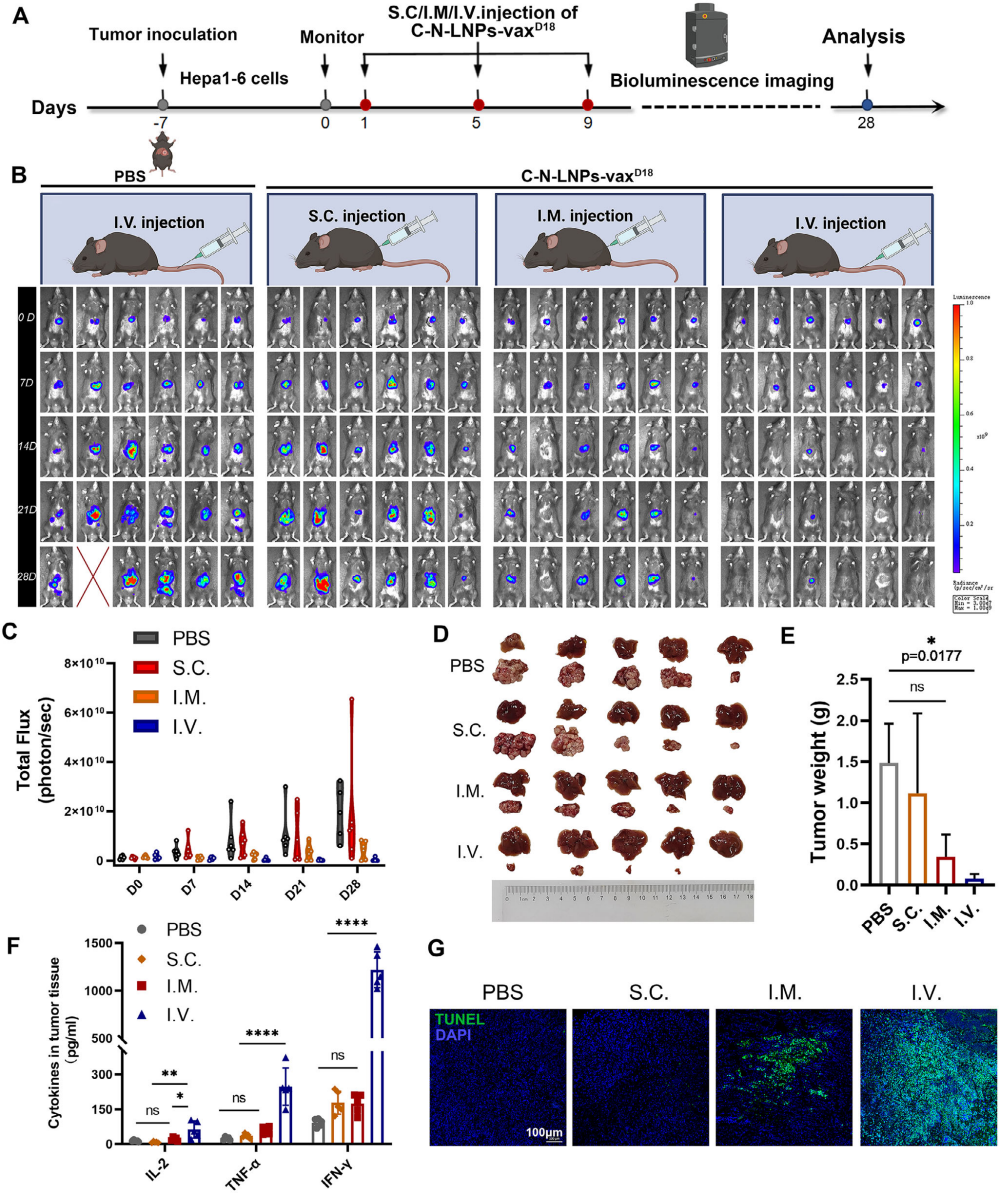
A) Diagram of the treatment schedule and analysis of established orthotopic Hepa1‐6‐luc tumor models after i.v. injection of PBS, s.c. injection of C‐N‐LNPs‐vax^D18^, i.m. injection of C‐N‐LNPs‐vax^D18^ or i.v. injection of C‐N‐LNPs‐vax^D18^ three times per four days. B) Representative bioluminescence imaging on days 0, 7, 14, 21, and 28 d post‐tumor inoculation, *n* = 6 after treatment as indicated in the established orthotopic Hepa1‐6‐luc bearing mice. Illustration created by BioRender.com. C) The total flux at 0, 7, 14, 21, and 28 d before and after receiving different treatments as indicated (*n* = 6). D) Image of ex tumor from orthotopic Hepa1‐6‐luc bearing mice and livers after indicated treatment (*n* = 5). E) The ex tumor weight in i.v. injection of PBS, s.c. injection of C‐N‐ LNPs‐vax^D18^, i.m. injection of C‐N‐LNPs‐vax^D18^, or i.v. injection of C‐N‐LNPs‐vax^D18^ treatment groups (*n* = 5). F) Cytokine levels of IFN‐γ, IL‐2, and TNF‐α in the tumors isolated from PBS, C‐N‐LNPs‐vax^D18^ with different administration strategies, which were determined by ELISA analysis (*n* = 5). G) TUNEL staining of tumors from orthotopic livers after receiving different treatments as indicated. Scale bar, 100 µm. Statistical analysis was performed with ANOVA analysis, ^*^p < 0.05, ^**^p < 0.01, ^***^p <* *0.001, ^****^p < 0.0001. Data are expressed as mean ± SD.

To validate our hypothesis, we employed an orthotopic liver tumor model to assess the antitumor efficacy of C‐N‐LNPs‐vax^D18^ through s.c., i.m., and i.v. injection (**Figure** **3**A). Bioluminescence imaging conducted 28 days post‐implantation revealed higher luciferase activity in the PBS‐treated group (Figure 3B,C). However, s.c. vaccination of mice did not exhibit significant therapeutic effects, possibly due to an insufficient antitumor immune response induced by local vaccine administration. This observation is consistent with previous studies of cancer vaccines in established tumor models.^[^ ^46^, ^47^ ^]^ In contrast, compared to s.c. vaccination, i.m. vaccination showed a mild reduction in tumor progression, as evidenced by the relatively lower luciferase intensity. This response could be attributed to the rich vascular system of the muscular tissue. Notably, the i.v. administration of the LNP‐neoantigen vaccine markedly suppressed tumor growth with minimal bioluminescence signals compared with s.c. or i.m. vaccination. These findings are further supported by ex tumor imaging of liver tissues and tumor weight, indicating that C‐N‐LNPs‐vax^D18^ exhibits superior therapeutic efficacy when administered intravenously compared with s.c. or i.m. vaccination (Figure 3D,E).

Furthermore, compared to administering C‐N‐LNPs‐vax^D18^ through s.c. or i.m. injection, ELISA analysis showed a significant increase in cytokine levels in tumor tissues after i.v. administration, including IFN‐γ, TNF‐α, and IL‐2 (Figure 3F). The elevated levels were approximately 13‐, 10‐, or 4.5‐fold, respectively, indicating the potential for a more robust induction of antitumor immunity by C‐N‐LNPs‐vax^D18^ via i.v. administration. In addition, TUNEL staining of the tumors confirmed the superior therapeutic efficacy of i.v. injection of C‐N‐LNPs‐vax^D18^, showing the largest area of green damage compared to the s.c. or i.m. injection of C‐N‐LNPs‐vax^D18^ (Figure 3G). These findings highlight that the intravenous injection of C‐N‐LNPs‐vax^D18^ could more effectively inhibit tumor growth.

### 2.4 I.V. Injection of C‐N‐LNPs‐vax^D18^ Modulates the Spleen and TME

**Figure 4:**
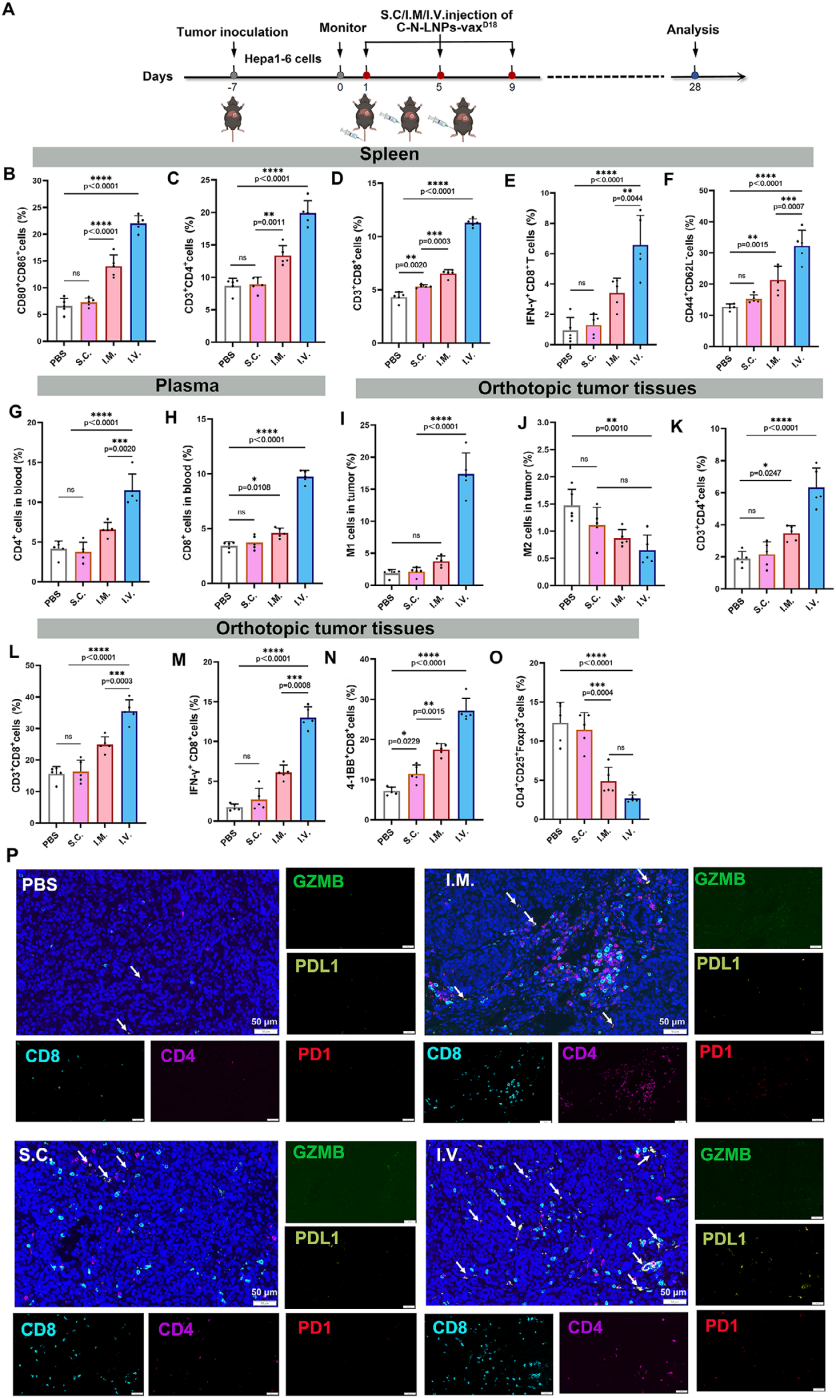
A) Diagram of the treatment schedule and analysis of immune response in established orthotopic Hepa1‐6‐luc tumor models after i.v. injection of PBS or C‐N‐LNPs‐vax^D18^ with different administration strategies three times per 4 days. Illustration created by BioRender.com. B) Percentage of CD80^+^CD86^+^ DCs cells, C) CD3^+^CD4^+^ T cells, D) CD3^+^CD8^+^ T cells, E) IFN‐γ^+^CD8^+^ T cells in the spleen after treatment as indicated (*n* = 5). F) The percentage of CD3^+^CD4^+^ T cells and G) CD3^+^CD8^+^ T cells in the blood after treatment as indicated (*n* = 5). I) Percentage of M1‐type TAMs that co‐expressed CD80, CD86, and CD11b and J) M2 TAMs that co‐expressed the CD206 and CD11b in tumors after treatments as indicated (*n* = 5). K) Percentage of infiltrated CD3^+^CD4^+^ T cells and L) CD3^+^CD8^+^ T cells, M) IFN‐γ^+^CD8^+^ T cells, N) 41BB^+^CD8^+^ T cells, and O) Foxp3^+^CD25^−^ Treg cells in tumors after treatments as indicated (*n* = 5). P) Multiplex immunofluorescence staining of the indicated markers for representative TILs in tumors after treatment as indicated. PDL1 expression (white arrows) in tumors. Scale bar, 50 µm. Statistical analysis was performed with ANOVA analysis, ^*^p < 0.05, ^**^p < 0.01, ^***^p < 0.001, ^****^p < 0.0001. Data are expressed as mean ± SD.

To study the immune response, we assessed DCs and T cells in the spleen, blood, and tumors in an orthotopic Hepa1‐6 liver tumor model after i.v. administration of C‐N‐LNPs‐vax^D18^ compared to the s.c. or i.m. administration (**Figure** **4**A). It was observed that i.v. vaccination led to a more efficient upregulation of co‐stimulatory receptor (CD80^+^ and CD86^+^) expression (22.6%) in splenic DCs compared to s.c. (7.66%) or i.m. (14.6%) vaccination with C‐N‐LNPs‐vax^D18^. This was attributed to the efficient accumulation and uptake of C‐N‐LNPs‐vax^D18^ in the spleen and DCs after i.v. administration (Figure 4B and Figure S11, Supporting Information). Moreover, the populations of CD4^+^ helper T cells (22.9%) and CD8^+^ effector T cells (11.8%), along with Tc1 (8.21%) in the spleen were substantially higher following i.v. vaccination with C‐N‐LNPs‐vax^D18^ compared to s.c. (CD4^+^ helper T cells, 9.89%; CD8^+^ effector T cells, 5.33% and Tc1, 1.63%) or i.m. (CD4^+^ helper T cells, 13.9%; CD8^+^ effector T cells, 6.67% and Tc1, 3.85%) vaccination (Figure 4C–E). In addition, i.v. administration of C‐N‐LNPs‐vax^D18^ resulted in stronger immune memory responses, as evidenced by the higher expression of co‐stimulatory receptors (CD44^+^CD62L^−^) in CD4^+^ memory T cells (32.26%) compared to s.c. injection (15.22%) or i.m. injection (21.36%) (Figure 4F). Similar results were observed in the blood, indicating that i.v. vaccination with C‐N‐LNPs‐vax^D18^ effectively triggered robust systemic and splenic T‐cell immune responses, whereas s.c. or i.m. vaccination was not as efficient in eliciting these responses (Figure 4G,H, and Figure S12, Supporting Information).

In contrast, TAMs are potential biomarkers of immunotherapeutic responses in different cancer types.^[^ ^48^ ^]^ We investigated the phenotype of TAMs, and the results showed that i.v. injection resulted in a higher frequency of M1‐type TAMs (17.38%) and significant infiltration of immune cells, such as CD4^+^ helper T cells (6.32%), CD8^+^ T cells (35.48%), Tc1 (13.00%), and 41BB^+^CD8^+^ T cells (27.18%), in tumor tissues (Figure 4I–N, Figure S13, Supporting Information). Conversely, the frequency of M2‐type TAMs (0.73%) decreased in the i.v. vaccination of C‐N‐LNPs‐vax^D18^‐treated groups compared to that in the s.c. (1.11%) or i.m. (0.87%) vaccination groups, potentially reducing M2‐type TAM‐induced immunosuppression. This effect is primarily attributed to the significant increase in IFN‐γ levels within tumors following i.v. administration of C‐N‐LNPs‐vax^D18^. The elevated IFN‐γ plays a crucial role in driving the differentiation of monocytes into proinflammatory M1 macrophages.^[^ ^49^ ^]^ These findings show a notable enhancement in immune responses within the “hot” TME with i.v. administration of C‐N‐LNPs‐vax^D18^ compared to s.c. injection (M1‐type TAMs, 2.12%; CD4^+^ helper T cells, 2.15%; CD8^+^ T cells, 16.34%; Tc1, 2.69%; 41BB^+^CD8^+^ T cells, 11.47%) or i.m. injection (M1‐type TAMs, 3.75%; CD4^+^ helper T cells, 3.47%; CD8^+^ T cells, 24.90%; Tc1, 6.11%; 41BB^+^CD8^+^ T cells,11.47%). The i.v. vaccination approach led to increased levels of M1‐type TAMs and various immune cells in tumor tissues, indicating a more favorable immune response profile for antitumor activity. Furthermore, the percentage of regulatory T cells (Tregs) in the group treated with C‐N‐LNPs‐vax^D18^ and receiving i.v. injection was only 2.65%, which was significantly lower than that observed in those who received s.c. (11.43%) or i.m. injections (4.88%) (Figure 4O). In addition, based on the tissue distribution of C‐N‐LNPs‐vax^D18^ after intravenous injection (Figure S14, Supporting Information), we showed that C‐N‐LNPs‐vax^D18^ first reached the lungs, then the liver, and finally, the spleen. We hypothesized that nanovaccines targeting tumors in the liver could polarize TAMs to the M1 phenotype, thereby remodeling the immunosuppressive TME. The accumulated C‐N‐LNPs‐vax^D18^ in the spleen can be taken up by APCs, initiating CD8^+^ T‐cell activation and promoting their migration and infiltration to tumor sites to enhance antitumor immunity. These results suggest that i.v. injection of C‐N‐LNPs‐vax^D18^ efficiently activates and enhances the tumor‐spleen immunity cycle by reducing the immunosuppressive TME.

Immunofluorescence analysis also confirmed higher infiltration of CD4^+^ and CD8^+^ T cells, along with increased M1‐type TAMs expressing CD86 and lower levels of CD206, in the tumors of mice that received intravenous injections of C‐N‐LNPs‐vax^D18^ compared to those that received s.c. or i.m. administration (Figure 4P and Figure S15, Supporting Information). These findings suggest that intravenous administration of C‐N‐LNPs‐vax^D18^ promotes the activation of T cells towards orthotopic tumors to boost cytotoxic T‐cell mediated antitumor immunity in the TME. Nonetheless, s.c. and i.m. vaccinations fail to effectively stimulate intratumoral T‐cell infiltration and tumor cell killing, likely because of inefficient accumulation of the vaccine in the tumor and spleen to initiate responses within the TME and spleen immunity.

**Figure 5:**
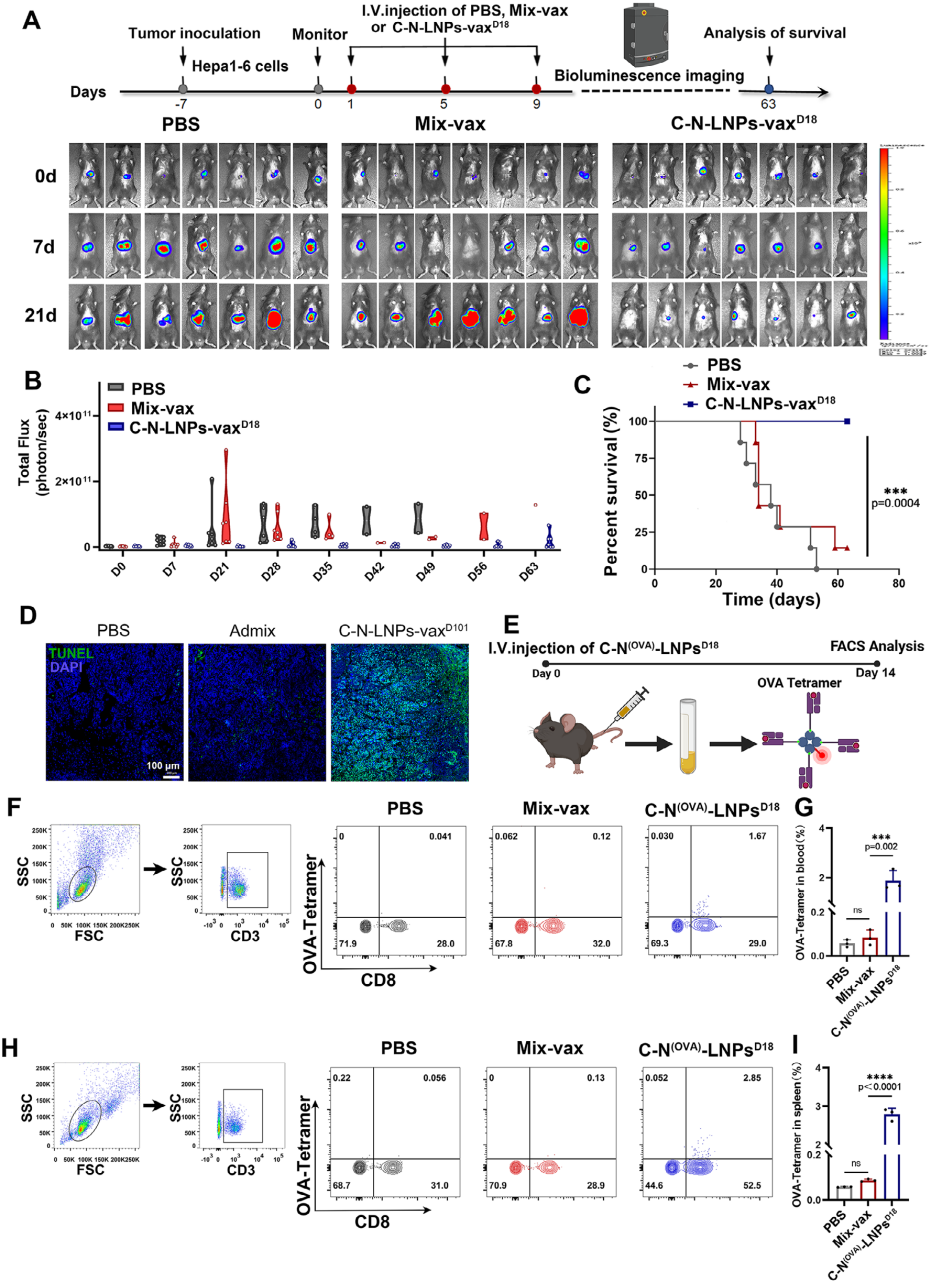
A) Diagram of treatment schedule and representative bioluminescence imaging on 0, 7, and 21 d, *n* = 7 after i.v. injection of PBS, Mix‐vax, or C‐N‐LNPs‐vax^D18^ in the established orthotopic Hepa1‐6‐luc bearing mice three times per four days. B) The total flux at 0, 7, 21, 28, 35, 42, 49, 56, and 63 d before and after receiving different treatments as indicated (*n* = 7). C) The survival curve of orthotopic Hepa1‐6‐luc bearing mice after indicated treatment (*n* = 7). Survival curves were calculated by using Kaplan–Meier estimates and the statistical analysis was performed with analysis of variance analysis. D) TUNEL staining of DNA breaks of tumor cells after receiving PBS, Mix‐vax, or C‐N‐LNPs‐vax^D18^ treatment in the established orthotopic Hepa1‐bearing mice. Scale bar, 100 µm. E) Schematic of OVA (SIINFEKL)^+^ specific CD8^+^ T cells in blood and spleen after receiving i.v. injection of PBS, Mix‐vax, or C‐N^(OVA)^‐LNPs^D18^. Illustration created by BioRender.com. F,G) FCM analysis showing the percentage of OVA (SIINFEKL)^+^ specific CD8^+^ T cells in the blood and H,I) spleen on days 14 (*n* = 3). Statistical analysis was performed with ANOVA analysis, ^*^p <* *0.05, ^**^p < 0.01, ^***^p < 0.001, ^****^p < 0.0001. Data are expressed as mean ± SD.

Next, we evaluated the therapeutic efficiency of C‐N‐LNPs‐vax^D18^ versus Mix‐vax through i.v. injection (**Figure** **5**A). On day 21, mice treated with PBS developed tumors with high luciferase activity. Similarly, mice treated with Mix‐vax showed minimal therapeutic efficacy and relatively higher luciferase activity (Figure 5B). In contrast, the mice treated with C‐N‐LNPs‐vax^D18^ exhibited lower tumor growth and significantly reduced luciferase activity by day 21 (Figure 5C and Figure S16, Supporting Information). Furthermore, treatment with C‐N‐LNPs‐vax^D18^ led to the complete elimination of tumors (2/7) in mice without any recurrence by day 63, whereas none of the PBS (0/7)‐ or Mix‐vax (0/7)‐treated mice showed tumor elimination. Notably, C‐N‐LNPs‐vax^D18^ effectively improved the overall survival of all mice (7/7) compared to PBS‐(0/7) or Mix‐vax‐treated mice (1/7). In addition, TUNEL staining of orthotopic tumors revealed a higher degree of cancer cell death in the C‐N‐LNPs‐vax^D18^ treatment group than in the Mix‐vax‐ or PBS‐treated groups, indicating the strong antitumor effect of C‐N‐LNPs‐vax^D18^ (Figure 5D). To certify neoantigen‐specific CD8^+^ T‐cell generation, mice were administered C‐N^OVA(SIINFEKL)^‐LNPs‐vax^D18^ on days 1, 5, and 9, and measured on day 14 using OVA‐specific tetramers (Figure 5E–I). The results demonstrated that C‐N‐LNPs‐vax^D18^ increased the frequency of OVA‐specific CD8^+^ T cells compared to the Mix‐vax‐ or PBS‐treated groups. Importantly, no significant weight loss or severe organ damage was observed in any of the mice after treatment (Figures S17 and S18, Supporting Information). These findings indicate that C‐N‐LNPs‐vax^D18^ is a safe and effective therapeutic vaccine against HCC.

To further compare the antitumor efficacy of the nanovaccines with and without the modification of DC‐targeted ligands, we evaluated the therapeutic performance of DC‐targeted C‐N‐LNPs‐vax^D18^ and nontargeted commercial liposome‐based nanovaccines (such as D‐Lin‐MC3‐DMA (MC3) and SM102) in orthotopic HCC tumor models (Figure S19, Supporting Information). The results showed that the residual tumor presented a relatively higher luciferase activity in mice treated with MC3‐ or SM102‐based vaccines than in those treated with C‐N‐LNPs‐vax^D18^. By day 7, the mice treated with PBS developed tumors with high luciferase activity, indicating rapid tumor growth. Similarly, mice treated with MC3‐ or SM102‐based neoantigen nanovaccines displayed a certain therapeutic efficacy, as evidenced by their relatively high luciferase activity and continued tumor growth. In contrast, mice treated with C‐N‐LNPs‐vax^D18^ showed significantly reduced tumor growth and luciferase activity on days 7, 14, and 21. Notably, 3/6 mice treated with C‐N‐LNPs‐vax^D18^ achieved complete tumor elimination, with no detectable tumors, compared to the MC3 (1/6)‐and SM102 (1/6)‐ based nanovaccine‐treated groups, where tumor presence was more prominent (images of the excised tumors from liver tissues). These results further highlighted the superior therapeutic efficacy of DC‐targeted C‐N‐LNPs‐vax^D18^ over nontargeted MC3‐ and SM102‐based vaccines. Additionally, T‐cell specific DNA‐binding protein (TCF1) regulates T‐cell development, and its expression in exhausted CD8^+^ T cells enhances antitumor immunity with stem cell‐like properties.^[^ ^50^ ^]^ We investigated the presence of TCF1^+^CD8^+^ T cells in tumor tissues after vaccination. The results demonstrated that compared to SM102‐ or MC3‐based vaccines (nontargeted nanovaccines), C‐N‐LNPs‐vax^D18^ significantly increased the number of TCF1^+^CD8^+^ T cells in tumors, resulting in enhanced therapeutic efficacy (Figure S20, Supporting Information). These findings highlight the enhanced APC‐targeting and antitumor activity of our formulation.

**Figure 6:**
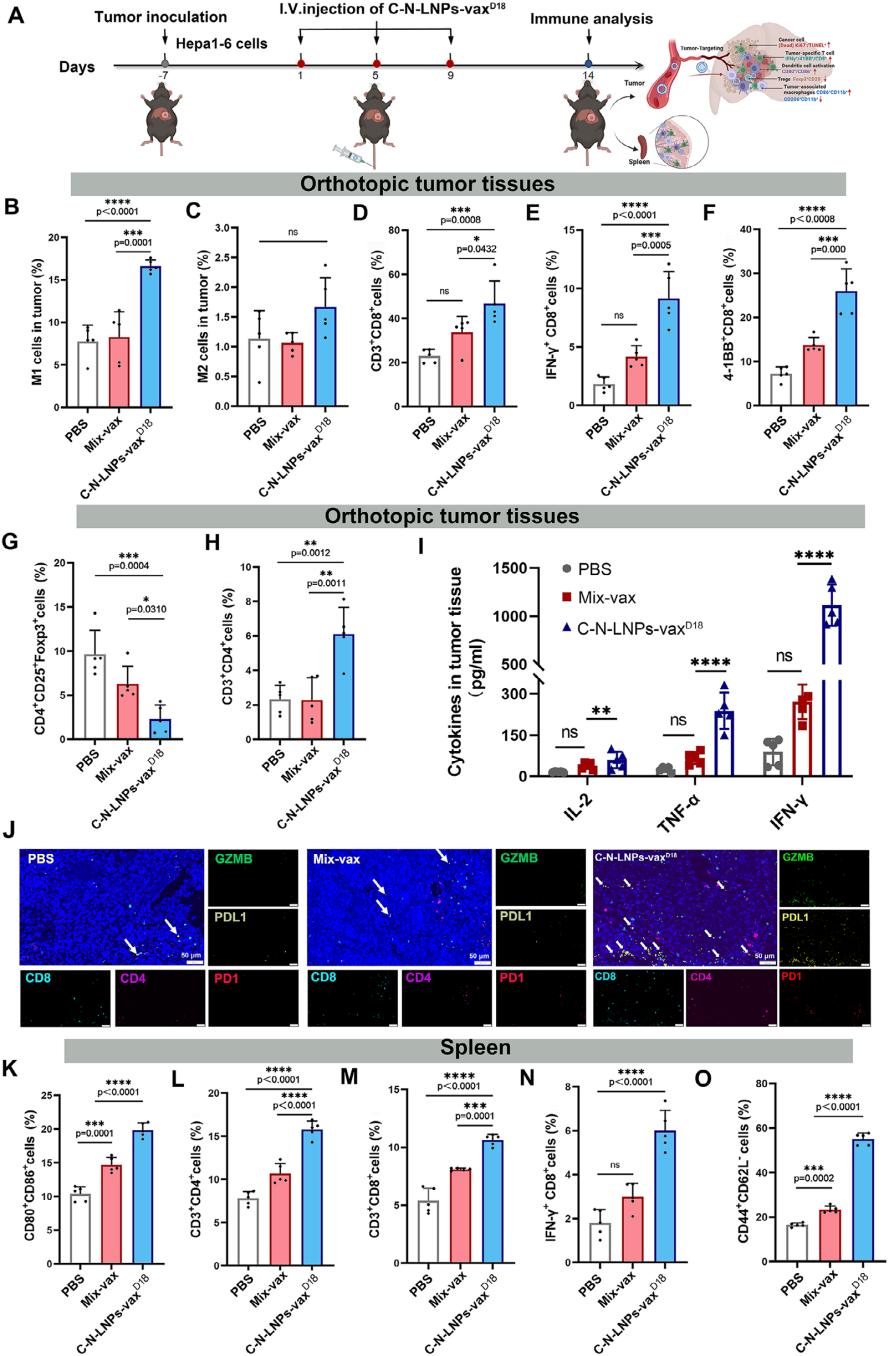
A) Diagram of treatment schedule and analysis of immune response in established orthotopic Hepa1‐6‐luc tumor models after i.v. injection of PBS, Mix‐vax, or C‐N‐LNPs‐vax^D18^ three times per four days. B) Tumors were harvested and analyzed by flow cytometry. Percentage of CD86^+^CD11b^+^ DCs cells, C) CD206^+^ CD11b^+^ DCs cells, D) CD3^+^CD8^+^ T cells, E) IFN‐γ^+^CD8^+^ T cells, 41BB^+^CD8^+^ T cells (F), Foxp3^+^CD25^−^ Treg cells (G), and CD3^+^CD4^+^ T cells (H) in the tumor tissues after treatment as indicated (*n* = 5). I) Cytokine levels of IFN‐γ, IL‐2, and TNF‐α in the tumor tissues were isolated from PBS, Mix‐vax, or C‐N‐LNPs‐vax^D18^ treated groups, which were measured by ELISA analysis (*n* = 5). J) Multiplex immunofluorescence for the indicated markers for representative TILs in tumors after treatment as indicated. PDL1 expression (white arrows) in tumors. Scale bar, 50 µm. K) Percentage of DCs that co‐expressed CD80 and CD86 in CD11c^+^ DC cells of the spleen after being treated with PBS, Mix‐vax, or C‐N‐LNP‐vax^D18^, and analyzed by flow cytometry, respectively (*n* = 5). L) Percentage of CD3^+^CD4^+^ T cells, M) CD8^+^CD3^+^ T cells, N) IFN‐γ^+^CD8^+^ T cells, and O) CD44^−^CD62L^+^memory T cells in the spleen after treatments as indicated (*n* = 5). Statistical analysis was performed with ANOVA analysis, ^*^p < 0.05, ^**^p < 0.01, ^***^p < 0.001, ^****^p < 0.0001. Data are expressed as mean ± SD.

To examine the status of the TiME after treatment, we analyzed the phenotypes of TAMs and CD11b, CD80, CD86, and CD206 expression after i.v. administration of C‐N‐LNPs‐vax^D18^ (**Figure** **6**A). A higher population of M1‐polarized TAMs (16.60%) was observed in tumors with C‐N‐LNPs‐vax^D18^ treatment compared to those treated with Mix‐vax (8.26%) (Figure 6B and Figure S21, Supporting Information). In contrast, the population of M2‐type TAMs did not differ significantly between the C‐N‐LNPs‐vax^D18^‐, Mix‐vax‐, and PBS‐treated groups (Figure 6C). These findings suggest that the targeted delivery of C‐N‐LNPs‐vax^D18^ helps shift TAMs to beneficial M1‐TAMs, thereby reducing the immunosuppressive effects of M2‐TAMs in the TME. In addition, immunofluorescence analysis confirmed a higher infiltration of M1‐type TAMs expressing CD86 and lower levels of CD206 in the tumors of mice that received C‐N‐LNPs‐vax^D18^ treatment compared to those that received PBS or Mix‐vax treatment (Figure S22, Supporting Information). Subsequently, the infiltration and characteristics of CD8^+^ T cells were investigated. Tumors treated with C‐N‐LNPs‐vax^D18^ showed higher percentages of CD8^+^CD3^+^ (46.66%), activated IFN‐γ^+^CD8^+^ (Tc1) (9.14%), and 41BB^+^CD8^+^ (25.96%) T cells compared to those treated with Mix‐vax (CD8^+^CD3^+^ T cells, 33.66%; IFN‐γ^+^CD8^+^ T cells, 4.17%; and 41BB^+^CD8^+^ T cells, 13.74%) and PBS (CD8^+^CD3^+^ T cells, 22.94%; IFN‐γ^+^CD8^+^ T cells, 1.82%; and 41BB^+^CD8^+^ T cells, 7.19%) treated groups (Figure 6D–F). Moreover, the population of Foxp3^+^CD25^+^ T cells (Tregs) in the tumors significantly decreased to 2.30% in mice treated with C‐N‐LNPs‐vax^D18^, which was notably lower than that in the PBS‐treated (9.65%) or Mix‐vax‐treated (6.29%) groups (Figure 6G). Moreover, the frequency of CD4^+^ helper T cells (6.12%) in the group treated with C‐N‐LNPs‐vax^D18^ was higher than that in groups treated with PBS (2.32%) or Mix‐vax (2.28%) (Figure 6H). Furthermore, ELISA analysis revealed a significant increase in IFN‐γ, TNF‐α, and IL‐2 cytokine levels in the tumor tissue following the administration of C‐N‐LNPs‐vax^D18^ compared to Mix‐vax or PBS treatment (Figure 6I). The increase was approximately 1.5‐4 times higher compared to the Mix‐vax treatment and 3.5‐12.5 times higher compared to the PBS treatment, leading to a more robust simulation of antitumor immunity and repression of tumor growth. These results suggest that C‐N‐LNPs‐vax^D18^ effectively modulates TiME, promoting the infiltration of antitumor CD8^+^ T cells and ultimately leading to the inhibition of tumor growth.

To further verify the vital role of CD4^+^ and CD8^+^ T cells in the C‐N‐LNPs‐vax^D18^ treatment, we depleted these cells using anti‐CD4 and anti‐CD8 antibodies (Figure S23, Supporting Information). The results demonstrated that the antitumor efficacy of C‐N‐LNPs‐vax^D18^ was significantly reduced in groups in which either CD4^+^ or CD8^+^ T cells were depleted compared to the group that received C‐N‐LNPs‐vax^D18^ treatment without T‐cell depletion. These findings highlighted the critical contributions of both CD4^+^ and CD8^+^ T cells in mediating the antitumor immune response elicited by C‐N‐LNPs‐vax^D18^. Furthermore, CD4^+^ and CD8^+^ T cells play complementary roles in C‐N‐LNPs‐vax^D18^ therapy. CD4^+^ T cells enhance the immune response by supporting CD8^+^ T‐cell activation and promoting a favorable TME. In turn, CD8^+^ T cells directly target and eliminate the tumor cells. The interplay between these two T‐cell populations is crucial for maximizing the therapeutic efficacy of C‐N‐LNPs‐vax^D18^ in established mouse tumor models and underscores the importance of T‐cell mediated immunity in cancer immunotherapy.

As TAMs can influence splenic immunity, the effect of C‐N‐LNPs‐vax^D18^ on the state of splenic DCs was assessed by harvesting splenic DCs and conducting FCM analysis (Figure 6J and Figure S24, Supporting Information). The results showed that C‐N‐LNPs‐vax^D18^ treatment efficiently enhanced the expression of co‐stimulatory receptors (CD80^+^ and CD86^+^) on CD11c^+^ DCs (19.84%) compared to Mix‐vax‐treated (14.68%) and PBS‐treated (10.37%) groups. Furthermore, the ability of DCs treated with C‐N‐LNPs‐vax^D18^ to activate naïve T cells was evaluated. A higher percentage of CD4^+^CD3^+^ helper (15.78%), CD3^+^CD8^+^ (10.63%), and IFN‐γ^+^CD8^+^ (6.00%) T cells were observed compared to the Mix‐vax‐treated groups (CD4^+^CD3^+^ T helper cells, 10.66%; CD3^+^CD8^+^ T cells, 8.09%; and IFN‐γ^+^CD8^+^ T cells, 2.99%) and PBS‐treated group (CD4^+^CD3^+^ T helper cells, 7.81%; CD3^+^CD8^+^ T cells, 5.39%; and IFN‐γ^+^CD8^+^ T cells, 1.80%) (Figure 6K–N). These findings suggested that C‐N‐LNPs‐vax^D18^ treatment enhanced T‐cell responses and efficiently activated splenic CD8^+^ T cells. Notably, C‐N‐LNPs‐vax^D18^ treatment induced strong central memory T cells (T_CM_), as evidenced by higher co‐stimulatory receptor expression (CD44^+^CD62L^−^) in CD8^+^ memory T cells (55.12%) compared to mice treated with Mix‐vax (23.34%) or PBS (16.46%) (Figure 6O). These findings demonstrated that C‐N‐LNPs‐vax^D18^ effectively regulated TAMs and splenic immune cell activation, including DCs and T cells, resulting in the generation of memory T cells that can help prevent tumor growth and relapse.

### 2.5 α‐PD1 Therapy Enhanced the Antitumor Effect of C‐N‐LNPs‐vax^D18^

**Figure 7:**
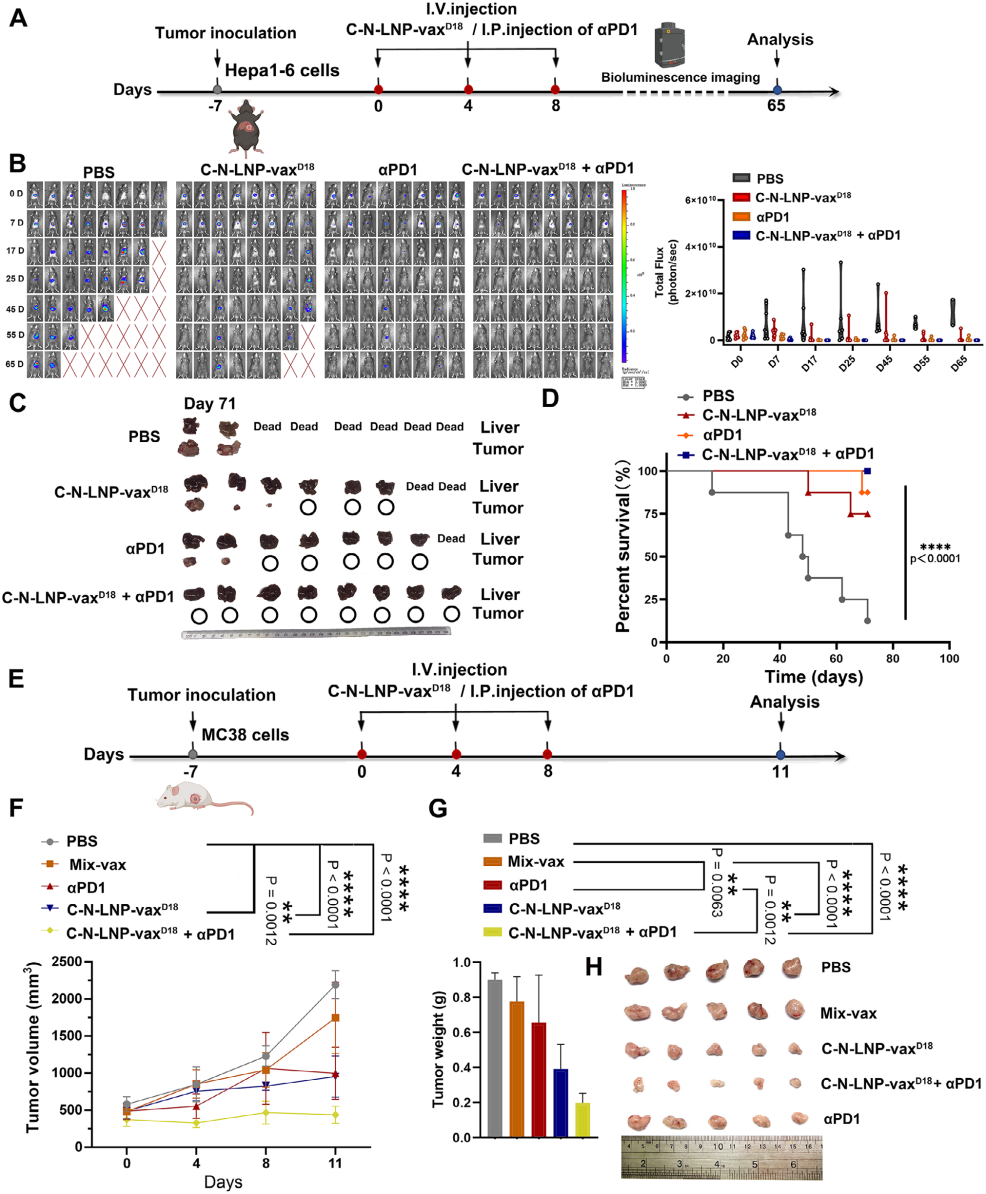
A) Diagram of the treatment schedule and B) representative bioluminescence imaging on 0, 7, 17, 25, 45, 55, and 65 d post‐tumor inoculation, *n* = 8 after i.v. injection of PBS, C‐N‐ LNP‐vax^D18^, αPD1 (i.p.), or C‐N‐LNPs‐vax^D18^ + αPD1 (i. p.) in the established orthotopic Hepa1‐6‐luc bearing mice for three times per 4 days. The total flux at 0, 7, 17, 25, 45, 55, and 65 d before and after receiving different treatments as indicated (*n* = 8). C) Ex tumor of liver cancer after different treatments as indicated. D) Photomicrograph of the ex tumor on day 71 after receiving different treatment as indicated (*n* = 8). E) Diagram of treatment schedule in established MC‐38 tumor models after i.v. injection of PBS, Mix‐vax, C‐N‐LNPs‐vax^D18^, αPD1 (i.p. injection) or C‐N‐LNPs‐vax^D18^ + αPD1 treated groups for three times per four days. F) Tumor volumes of MC‐38 bearing BALb/c mice treated with PBS, Mix‐vax, C‐N‐LNPs‐vax^D18^, αPD1 (i.p. injection) or C‐N‐LNPs‐vax^D18^ + αPD1, respectively (*n* = 5 mice per group; data are shown as means ± SD). G) Ex tumor weights of mice on day 11 after receiving different treatments as indicated (*n* = 5). H) Photomicrograph of ex tumor after the indicated treatments (*n* = 5). Statistical analysis was performed using ANOVA analysis, ^*^p < 0.05, ^**^p < 0.01, ^***^p < 0.001, ^****^p < 0.0001. Data are expressed as mean ± SD. Illustration created by BioRender.com.

Our previous findings indicated the consistent overexpression of immune checkpoints in tumors after vaccination.^[^ ^10^, ^51^ ^]^ Therefore, we further investigated whether a combination of intraperitoneal injection (i.p.) of a PD1 inhibitor and i.v. injection of C‐N‐LNPs‐vax^D18^ enhanced therapeutic efficiency and prolonged progression‐free survival (PFS). (**Figure** **7**A). After i.v. vaccination of C‐N‐LNPs‐vax^D18^ and i.p. injection of α‐PD1 antibody three times every 4 days, bioluminescence imaging conducted at day 65 post‐implantation showed higher luciferase activity in the PBS‐treated group (Figure 7B). However, treatment with α‐PD1 alone or C‐N‐LNPs‐vax^D18^ alone showed significant inhibition of tumor growth, as evidenced by relatively lower luciferase activity on day 65. However, the tumor was not eliminated. When combined with α‐PD1 therapy and C‐N‐LNPs‐vax^D18^ vaccination, there was a significant inhibition of tumor growth without any luciferase activity on day 65. This combination achieved higher complete response (CR) in mice (CR, 8/8) compared to PBS‐ (CR, 0/8), C‐N‐LNPs‐vax^D18^‐ (CR, 3/8), and α‐PD1‐treated (CR, 5/8) groups on the day 71 (Figure 7C,D). This combination therapy shows promise in enhancing immune responses against cancer and improving treatment outcomes.

**Figure 8:**
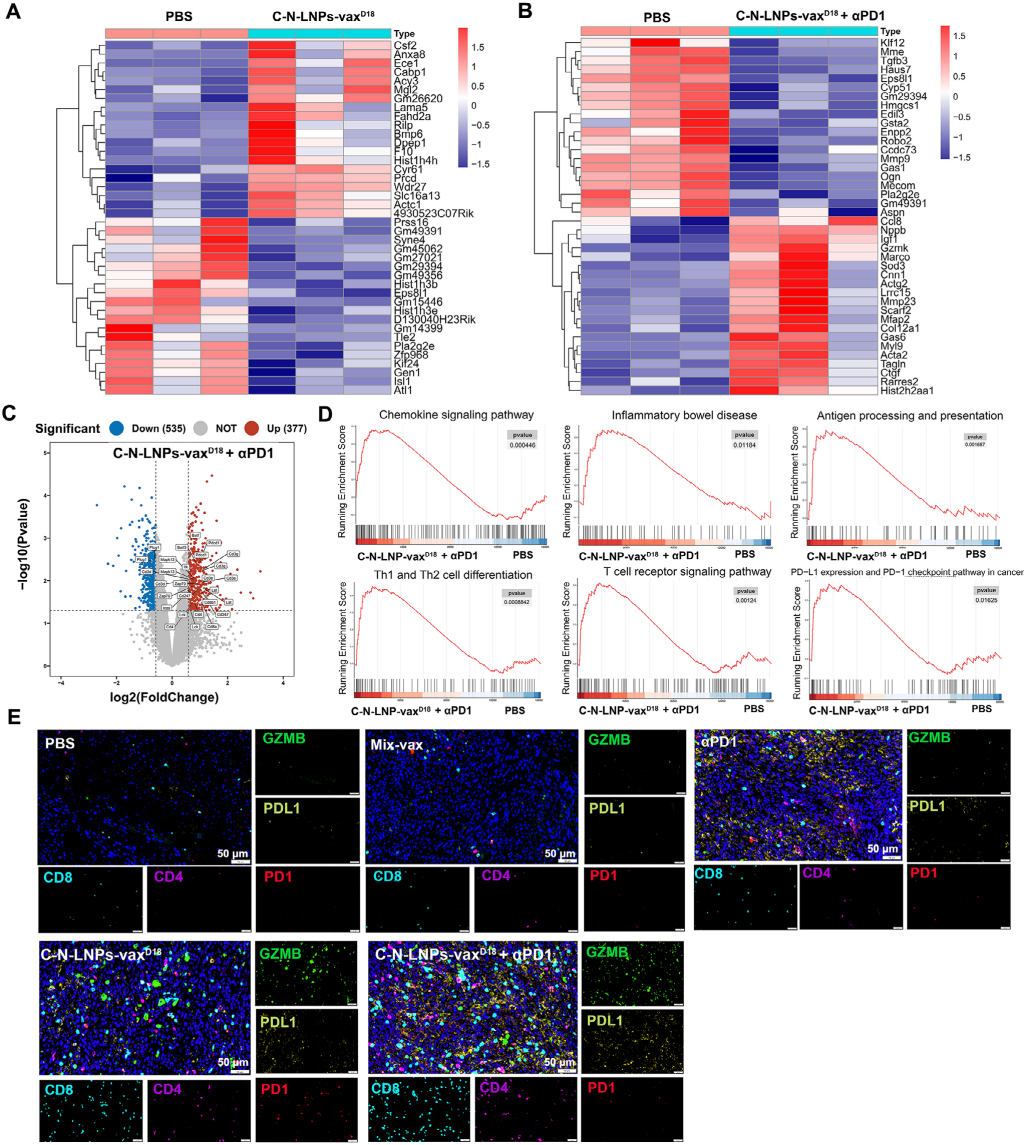
A,B) Heat map of RNA‐seq results of tumors with the combinational treatment of C‐N‐LNPs‐vax^D18^ or/and αPD1 compared to untreated tumors (PBS group). The top 20 upregulated functional pathways in the combinational treatment of C‐N‐LNPs‐vax^D18^ and αPD1 vs. PBS‐treated group, as determined by KEGG analysis. C) Volcano plot showing the upregulated, unchanged, or downregulated genes when comparing the C‐N‐LNPs‐vax^D18^ and αPD1‐treated mice with PBS‐treated group. D) Gene set enrichment analysis (GSEA) is based on differentially expressed genes between the C‐N‐LNPs‐vax^D18^ + αPD1 and the PBS group (*n* = 3 mice per group). E) Multiplex immunofluorescence for the indicated markers for representative TLSs in tumors after treatment as indicated. Scale bar, 50 µm.

Building on the aforementioned results, additional testing of the LNP‐vax platform in the MC38 established tumor model was conducted to confirm its significant antitumor efficacy (Figure 7E). Integration of MC38 tumor‐specific neoantigens into C‐N^MC38^ (GNNAFRVYLMLPLSERP)‐LNPs‐vax^D18^ resulted in significant suppression of MC38 tumor progression compared to Mix‐vax‐treated groups (Figure 7F). The C‐N^MC38^‐LNPs‐vax^D18^ treatment alone resulted in a notable reduction in tumor size and weight, as confirmed by ex vivo tumor weight and imaging (Figure 7G). The therapeutic efficiency was comparable to that of α‐PD1 therapy alone. However, when combined with α‐PD1 therapy and C‐N^MC38^‐LNPs‐vax^D18^, the antitumor effect was significantly improved in MC38 tumor mouse models, resulting in the lowest tumor weights and volumes compared to α‐PD1 therapy or C‐N^MC38^‐LNPs‐vax^D18^ treatment alone. To further study alterations in the transcription of genes following combined therapy, we conducted gene ontology and biological process analyses to examine the mechanisms and signals involved in the therapeutic responses (**Figure** **8**A,B). Gene set enrichment analysis (GSEA) showed that treatment with C‐N^MC38^‐ LNPs‐vax^D18^ and α‐PD1 therapy led to increased activation of various pathways including chemokine signaling, inflammatory, antigen processing, and presentation, Th1 and Th2 cell differentiation, T‐cell signaling, and the α‐PD1 checkpoint pathways. The upregulation of chemokines, particularly CCL8, facilitated the infiltration of immune cells such as monocytes, lymphocytes, basophils, and eosinophils into tumors, leading to remodeling of the TiME (Figure 8C,D, and Figure S25, Supporting Information). Immunofluorescence analysis further confirmed higher PD1^+^CD8^+^ T cell and GZMB^+^CD8^+^ T‐cell infiltration in tumors of mice that received combination treatment of C‐N^MC38^‐LNPs‐vax^D18^ and α‐PD1 in comparison to those that received PBS, Mix‐vax, α‐PD1, or C‐N^MC38^‐LNPs‐vax^D18^ treatment alone (Figure 8E). Moreover, combination therapy increased M1‐type TAMs with relatively higher CD86 and lower CD206 levels in tumors, further corroborating the above results (Figure S26, Supporting Information). Overall, combination therapy induced significant accumulation of immune effector cells in orthotopic tumors, highlighting its potential to enhance antitumor immune responses.

## 3 Discussion

Compared to previous DC‐targeting vaccines that relied on ligand modifications,^[^ ^36^ ^]^ our study demonstrated highly efficient targeting and uptake of the LNPs^D18^ formulation by APCs in the spleen and tumors without requiring additional ligand modification. This formulation, when used as a neoantigen peptide vaccine, significantly enhanced the adaptive antitumor immunity against established solid tumors. The screening of the LNPs^D18^ formulations was guided by the critical role of APCs in antigen cross‐presentation. Although there is a significant body of research dedicated to screening organ‐targeting LNP formulations,^[^ ^37^, ^52^, ^53^ ^]^ limited attention has been paid to APC‐targeting LNPs in tumors and the spleen.^[^ ^33^, ^54^ ^]^ A key discovery was the significantly enhanced efficacy of C‐N‐LNPs‐vax^D18^ in APC internalization both in vitro and in vivo, possibly because of the enhanced uptake of APCs by cationic dimethyldioctadecylammonium‐based liposomes.^[^ ^55^ ^]^ Additionally, C‐N‐LNPs‐vax^D18^ exhibited targeting the splenic DCs, macrophages, and TAMs. This facilitated the remodeling of the TiME by boosting M1‐type TAMs while reducing Tregs in tumor tissues compared to previous LNP formulations.^[^ ^56^, ^57^ ^]^ Notably, the frequency of M2‐TAMs was not reduced in the C‐N‐LNPs‐vax^D18^ treated groups. This increase in the population of M1‐TAMs may be linked to earlier observations indicating that CpG‐ODNs can drive MDSCs to mature into M1 macrophages.^[^ ^16^ ^]^ In addition, C‐N‐LNPs‐vax^D18^ induced CD8^+^ T cells to adopt a T_CM_ central memory phenotype associated with long‐term tumor control. Through their synergistic effects on the TAM phenotype, DC antigen presentation, and the population of Tc1 in tumors, C‐N‐LNPs‐vax^D18^ effectively inhibited orthotopic tumor growth and prolonged survival in an established HCC model.

Intravenous injection of peptide nanovaccines could enhance the population of stem‐like TCF1^+^CD8^+^ T cells to improve their antitumor immunity compared with s.c. immunization.^[^ ^45^ ^]^ In the present study, we propose an optimized approach to efficiently stimulate the tumor‐spleen immunity cycle to inhibit established mouse tumor models, which also involves i.v. injection of C‐N‐LNPs‐vax^D18^. Compared to s.c. or i.m. vaccination, i.v. administration resulted in an increased frequency of Tc1 and CD4^+^ T helper cells in the tumor and spleen, as well as a higher ratio of CD8^+^ T cells to CD4^+^ T helper cells in the blood, suggesting successful T‐cell activation. The i.v. vaccination also led to an increase in 41BB^+^CD8^+^ and TCF1^+^ CD8^+^ T cells, which are known for their efficient proliferation, effector functions, and enhanced antitumor immunity with stem cell‐like properties. The efficient delivery of neoantigens and CHO‐CpG to the spleen and tumors through i.v. administration of LNPs^D18^ is responsible for this advance, which significantly promotes systemic immunity. Combining immune checkpoint blockade inhibitors such as αPD1 could further enhance therapeutic efficiency.

In summary, this study introduces APC‐targeting LNP^D18^ that administers neoantigens and adjuvants to the same DC using liposome nanoparticles. By targeting the activation of splenic DCs, macrophages, and tumors, this vaccine remodels the TiME by reducing Tregs and enhancing M1‐TAM populations in tumors. This leads to significant antitumor responses from activated CD8^+^ and TCF1^+^ CD8^+^ T cells, inhibiting tumor growth via intravenous injection instead of conventional s.c. or i.m. administration of the LNP‐neoantigen vaccine. These findings offer valuable insights for the development of an LNP‐based vaccine as a novel cancer immunotherapy that could improve the prognosis of HCC or CRC. These findings have the potential to advance the development of therapeutic vaccines.

## 4 Experimental Section

### Materials

Dimethyl dioctadecylammonium bromide (DDAB) was purchased from Sigma‐Aldrich (Germany). 1, 2‐di‐*O*‐octadecenyl‐3‐trimethylammonium propane (DOTMA), 1,2‐dioleoyl‐sn‐glycero‐3‐phosphoethanolamine (DOPE), and cholesterol (CHO) were purchased from AVT Pharmaceutical Technology (Shanghai, China). The DiR fluorescent dye was obtained from Thermo Fisher Scientific (USA). CCK‐8 assay kits were purchased from Dojindo Laboratories (Shanghai, China). Anti‐CD11c‐APC, anti‐CD80‐PE, anti‐CD86‐PE‐Cy7, anti‐CD4‐FITC, anti‐CD8‐PE, anti‐CD3‐APC, anti‐CD44‐PE‐Cy7, anti‐CD62L‐Percp‐Cy5.5, anti‐CD11b‐APC, anti‐CD206‐PE, anti‐Foxp3‐PE‐Cy7, anti‐IFN‐γ‐PE‐Cy7, anti‐CD206‐PE, anti‐CD3‐FITC, anti‐4‐1BB‐APC anti‐CD25‐Percp‐Cy5.5, anti‐CD19‐FITC, anti‐F4/80‐PE, and anti‐CD3‐APC were purchased from BioLegend (San Diego, CA, USA). Anti‐CD205 (BioCell), anti‐CD4 (Leinco), and anti‐CD8 (Leinco) antibodies were purchased from Leinco and Biocell. Anti‐TCF1 (Alexa Fluor 700) antibody was purchased from R&D Systems (USA).

ELISA kits for detecting IFN‐γ, TNF‐α, and IL‐2 were purchased from Fankewei (Shanghai, China). Mouse liver cancer Hepa1‐6 cell‐specific neoantigens (WDTCTTYKWQKTLEGHD and WDTCTTYKWQKTLEGHD‐FAM) and MC38 cell‐specific neoantigens (GNNAFRVYLMLPLS ERP) were identified in the previous studies, and detailed screening methods can be found in the previous protocol.^[^ ^58^, ^59^ ^]^ Neoantigens were synthesized using GenScript (USA). Cholesterol‐coupled thio‐modified CpG oligodeoxynucleotides (CHO‐CpG), (T*C*C*A*T*G*A*C*G*T* T*C*C*T*G*A*C*G*T*T*‐CHO, *represent thio‐modification base), (CHO‐^Cy5^CpG: Cy5‐T*C *C*A*T*G*A*C*G*T*T*C*C*T*G*A*C*G*T*T*‐CHO) were synthesized by Sango Biotechnology, China.

### Cell Culture and Animals

Mouse Hepa1‐6 liver cancer cells, Hepa1‐6‐luc cells stably expressing luciferase (luc), and the mouse MC38 colorectal cancer cell line were cultured in DMEM with 100 IU mL^−1^ penicillin‐streptomycin and 10% FBS (v/v) at 37 °C. Female C57BL/6 mice (4–5 weeks old) were procured from China Wushi (Shanghai, China). Animal care and procedures were conducted according to the Guide for the Care and Use of Laboratory Animals. The Animal Ethics Committee of the Mengchao Hepatobiliary Hospital of Fujian Medical University approved all animal protocols. All animal procedures were approved by the Animal Ethics Committee of Mengchao Hepatobiliary Hospital of Fujian Medical University (MCHH‐AEC‐2023‐03‐03).

### Synthesis of Liposome‐Neoantigen Vaccines

To prepare the LNP‐neoantigen vaccines (C‐N‐LNPs‐vax), a panel of LNPs was initially synthesized by using an ethanol injection method followed by extrusion through a 0.25 µm membrane filter. By employing specific molar ratios of DOTMA, DDAB, DOPE, and CHO, a series of LNPs were obtained named LNPs^D18^ (36.36% DOTMA, 36.36% DOPE, 9.09% CHO, and 18.18% DDAB), LNPs^D30^ (30.77% DOTMA, 30.77% DOPE, 7.69% CHO, and 30.77% DDAB), LNPs^D47^ (23.53% DOTMA, 23.53% DOPE, 5.88% CHO, and 47.06% DDAB), LNPs^D64^(16% DOTMA, 16% DOPE, 4% CHO, and 64% DDAB), LNPs^D72^ (12.12% DOTMA, 12.12% DOPE, 3.03% CHO, and 72.73% DDAB), and LNP‐vax^D78^ (9.77% DOTMA, 9.77% DOPE, 2.44% CHO, and 78.05% DDAB). Then, predetermined amounts of Hepa1‐6 tumor‐specific or MC38 tumor‐specific neoantigens and CHO‐CpG were added to the LNP^D18^, LNP^D30^, LNP^D47^, LNP^D64^, LNP^D72^, and LNP^D78^ solutions and stirred for 2–4 h. The obtained C‐N‐LNPs‐vax was purified by dialysis (14 KDa) to eliminate organic solvents and excess neoantigens. The obtained C‐N‐LNPs‐vax formulations, designated as C‐N‐LNPs‐vax^D18^ (33.45% DOTMA, 33.45% DOPE, 8.36% CHO, 16.72% DDAB, 0.17% CHO‐CpG, and 7.86% neoantigen), C‐N‐LNPs‐vax^D30^(28.65% DOTMA, 28.65% DOPE, 7.16% CHO and 28.65% DDAB, 0.14% CHO‐CpG, and 6.73% neoantigen), C‐N‐LNPs‐vax^D47^ (22.27% DOTMA, 22.27% DOPE, 5.57% CHO, 44.54% DDAB, 0.11% CHO‐CpG, and 5.23% neoantigen), C‐N‐LNPs‐vax^D64^ (15.41% DOTMA, 15.41% DOPE, 3.85%CHO, 61.63% DDAB, 0.08% CHO‐CpG, and 3.62% neoantigen), C‐N‐LNPs‐vax^D72^ (11.78% DOTMA, 11.78% DOPE, 2.95% CHO, 70.67% DDAB, 0.06% CHO‐CpG, and 2.77% neoantigen), and C‐N‐LNPs‐vax^D78^ (9.53% DOTMA, 9.53% DOPE, 2.38% CHO, 76.26% DDAB, 0.05% CHO‐CpG, and 2.24% neoantigen), were stored at 4 °C for further use. To prepare SM102 or D‐Lin‐MC3‐DMA (MC3) LNP‐neoantigen vaccines, the molar ratios of SM102 or MC3, DSPE, CHO, and DMG‐PEG were 50:10:38.5:1.5 according to previous studies.^[^ ^60^, ^61^ ^]^ Then, a predetermined amount of Hepa1‐6 tumor‐specific or MC38 tumor‐specific neoantigens and CpG‐ODNs was added to the SM102‐LNPs, or MC3‐LNPs solution and stirred for 2–4 h. The obtained SM102‐LNP‐vax and MC3‐LNP‐vax were purified by dialysis (14 KDa) to eliminate organic solvents and excess neoantigens. A standard curve was established, demonstrating excellent linearity with neoantigen concentrations ranging from 0 to 500 µg mL^−1^ (*Y* = 5.5039*x* + 70.614, *R* ^2^ = 0.9935). Similarly, the linear fit for CHO‐^Cy5^CpG concentrations in the range of 0–2.5 nmol was *Y* = 105559*x* – 0.293, *R* ^2^ = 0.9994. The quantities of ^FAM^Neoantigen and CHO‐ ^Cy5^CpG in C‐N‐LNPs‐vax were determined by measuring the UV–Vis absorbance of the supernatants and comparing the readings to the corresponding standard curves. The doses of neoantigen and CHO‐CpG in C‐N‐LNPs‐vax^D18^, C‐N‐LNPs‐vax^D30^, C‐N‐LNPs‐vax^D47^, C‐N‐LNPs‐vax^D64^, C‐N‐LNPs‐vax^D72^, and C‐N‐LNPs‐vax^D78^ were approximately 50.0 µg/160 µg and 3.20 µg/160 µg, respectively. The loading efficiency was determined to be 2 wt% for CHO‐CpG and 23.45 wt% for the peptide in C‐N‐LNPs‐vax^D18^.

### Apparatus

Flow cytometric analysis was conducted using a BD FACSVerse system. CLSM was performed using a ZEISS LSM 780. The structure of C‐N‐LNPs‐vax^D18^ was examined using cryogenic electron microscopy (cryo‐EM) with an FEI Tecnai G20 microscope. A Zetasizer NanoZS (ZEN3600) was used to determine the nanoparticle size and zeta potential of LNPs^D18^ and C‐N‐LNPs‐vax^D18^. Absorbance spectra for the LNPs^D18^ and C‐N‐LNPs‐vax^D18^ were recorded by Spectro Max M5e.

### Cellular Uptake of Liposome‐Neoantigen Vaccines by DC2.4 Cells

To assess the cellular uptake of C‐N‐LNPs‐vax^D18^, C‐N‐LNPs‐vax^D30^, C‐N‐LNPs‐vax^D47^, C‐N‐LNPs‐vax^D64^, C‐N‐LNPs‐vax^D72^, and C‐N‐LNPs‐vax^D78^, DC2.4 cells were cultured at a density of 1 × 10^5^ cells per well on 35 mm Petri dishes with glass bottom and maintained at 37 °C for 24 h. Afterward, the cells were exposed to C‐N‐LNPs‐vax^D18^, C‐N‐LNPs‐vax^D30^, C‐N‐LNPs‐vax^D47^, C‐N‐LNPs‐vax^D64^, C‐N‐LNPs‐vax^D72^, and C‐N‐LNPs‐vax^D78^ labeled with Dir dye for 12 h. Subsequently, the nucleus was stained using DAPI excited at 405 nm, and the cells were washed once with PBS (pH 7.4) and imaged using CLSM or quantified using FCM. To investigate the cellular uptake of CHO‐ ^Cy5^CpG, ^FAM^neoantigen DC2.4 cells were cultured at a concentration of 1 × 10^5^ cells per well on 35 mm glass‐bottom dishes. Subsequently, the DC2.4 cells were maintained at 37 °C for 24 h and treated with C‐N‐LNPs‐vax^D18^ for 12 h at 37 °C. Finally, the nuclei were stained with DAPI at 405 nm, and the treated cells were imaged using CLSM or quantified by FCM. FAM fluorescence was excited at 488 nm and Cy5 fluorescence was excited at 633 nm. To evaluate the cellular uptake efficiency of C‐N‐LNPs‐vax^D18^ by BMDCs with or without blocking by anti‐CD205 antibody, BMDCs were cultured at a density of 1 × 10^5^ cells per well in 35 mm glass‐bottom Petri dishes and maintained at 37 °C for 24 h. Afterward, the cells were treated with an anti‐CD205 antibody (10 µg mL^−1^) for 12 h, then co‐incubated with Dir dye‐labeled C‐N‐LNPs‐vax^D18^ for 12 h. The treated cells were analyzed and quantified by FCM.

### Potential Toxicity of Liposome‐Neoantigen Vaccines In Vitro and In Vivo

To assess the cytotoxicity of C‐N‐LNPs‐vax^D18^, the DC2.4 cells were seeded at 1 × 10^5^ cells per well in 96‐well plates and incubated for 24 h. Subsequently, the cells were exposed to varying concentrations of C‐N‐LNPs‐vax^D18^, ranging from 0 to 100 µg mL^−1^, for an additional 24 or 48 h in fresh medium. Cell viability was evaluated using CCK‐8 assays.^[^ ^46^ ^]^ To evaluate the systemic toxicity of C‐N‐LNPs‐vax^D18^, healthy female C57BL/6 mice (4–5 weeks) were i.v. administrated with C‐N‐LNPs‐vax^D18^ (213.5 µg). Blood was collected for analysis, and the major organs (heart, liver, spleen, lungs, and kidney) of the mice were collected, fixed in 4% neutral formaldehyde, embedded in paraffin, stained with hematoxylin and eosin (H&E), and observed under a Zeiss microscope (Axio Lab.A1).

### Western Blotting Analysis

Cell lysates were prepared using RIPA lysis buffer solution (Beyotime Biotechnology, China) with PMSF and a protease inhibitor cocktail (MedChemExpress, China). The protein content of CHO cell lysates was determined using the BCA protein assay (TransGen Biotech, China). The aliquots with 30 µg of total protein were loaded onto SDS‐polyacrylamide gels and transferred to nitrocellulose membranes. Following blocking the membranes with 5% BSA for 1 h, the membranes were probed overnight at 4 °C with the indicated primary antibodies, anti‐CD205 (21719, Santa Cruz) and anti‐β‐actin (3P8, Abmart), followed by incubation with HRP‐conjugated secondary antibodies (Abcam, 1:5000) for 1 h at 21 °C.

### BMDC Maturation

BMDC maturation was analyzed following a previously published procedure.^[^ ^34^ ^]^ Tibias and femurs were obtained from female C57BL/6 mice aged 4‐5 weeks, kept on ice in the medium. The bones were trimmed with scissors and the cells were flushed with medium to isolate any remaining cells. The cells were filtered with a 40 µm strainer to eliminate debris, centrifuged, and resuspended in red blood cell lysis buffer to lyse red blood cells. After collection, washing, and centrifugation, the cells were cultured in a medium containing 20 ng mL^−1^ mouse granulocyte/macrophage colony‐stimulating factor (mGM‐CSF) and 10 ng mL^−1^ IL‐4 within 6‐well plates. After 5 days, the inactive BMDCs were treated with a combination of 5 µg neoantigen and 0.034 µg CpG (refer to Mix‐vax), or C‐N‐LNPs‐vax^D18^ (5 µg neoantigen and 0.034 µg CHO‐CpG) for 48 h. BMDCs were stained using eBioscience antibodies against CD11c, CD80, and CD86. FCM was used to analyze the results.

### Cytokine Analysis of Tumor Lysate

On day 14, tumor tissues were harvested and isolated from the mice following vaccination. Thirty milligrams of tumor tissue were collected and lysed in RIPA lysis buffer (Beyotime Biotechnology, China) containing a protease inhibitor cocktail (MedChem Express, China). The tumor tissues were homogenized with 5 mm magnetic beads at 50 Hz for 6 min and then centrifuged at 15 000 × *g* for 6 min at 4 °C. The obtained supernatants were tested for IFN‐γ, TNF‐α, or IL‐2 using ELISA (Boster Biological Technology, USA) following the manufacturer's instructions.

### In Vitro Re‐Stimulation for IFN‐γ and Enzyme‐Linked Immunospot (ELISpot) Assay

Female C57BL/6 mice, aged from 6 to 8 weeks, were acquired from China Wushi (Shanghai, China). To explore the immunogenicity of C‐N‐LNPs‐vax^D18^, these vaccines or a mixture of neoantigen and CpG‐ODN were used for i.v. injection of the female C57BL/6 mice on days 1, 5, and 9. The mice were euthanized on day 14, and splenic T cells were harvested and counted for the ELISPOT assay. IFN‐γ secretion of mouse splenic T cells were detected using an ELISPOT kit (Mabtech, 3321–4APT‐10). Detailed methods are described in the previous report.^[^ ^10^ ^]^

### Histological Evaluation

Tumors were harvested on day 63 post‐treatment and processed for histological analysis. H&E staining was performed, along with immunohistochemical staining for Ki67 and TUNEL (R&D Systems). In addition, the tumor sections were subjected to immunofluorescence staining for CD4 and CD8 (Servicebio Systems).

### Biodistribution of C‐N‐LNPs‐vax^D18^

To assess the distribution of C‐N‐LNPs‐vax^D18^, Hepa1‐6‐luc tumor‐bearing female C57BL/6 mice were injected intravenously with Dir labeled C‐N‐LNPs‐vax^D18^ containing 50 µg FAM‐labeled neoantigen and 3.2 µg CHO‐CpG. The liver, tumor, spleen, lung, kidney, and heart were isolated and imaged using an IVIS Lumina X5 after 24 h. Spleen tissues were homogenized using a nylon filter. Harvested cells were individually stained with anti‐CD11c‐APC, anti‐CD19‐FITC, anti‐F4/80‐PE, anti‐CD3‐APC, and anti‐NK1.1‐PE‐Cy7 antibodies for 30 min, followed by analysis via FCM to determine the number of C‐N‐^Dir^LNP‐vax^D18^‐positive cells. These cells were used for FCM analysis.

### Antitumor Immunotherapeutic Effect of C‐N‐LNPs‐vax^D18^ In Vivo

To assess the antitumor efficacy of C‐N‐LNPs‐vax^D18^, female C57BL/6 mice aged 4–5 weeks were obtained from China Wushi (Shanghai, China). Animal experiments were performed according to procedures approved by the Animal Ethics Committee of the Mengchao Hepatobiliary Hospital of Fujian Medical University (MCHH‐AEC‐2023‐11). The mice were injected with Hepa1‐6‐Luc cells (3.5 × 10^5^) directly under the liver capsule. After 7 days, the successful establishment of orthotopic Hepa1‐6 liver tumors was confirmed using IVIS Lumina X5. The tumor‐bearing mice were randomly assigned to one of the following treatment groups: PBS, Mix‐vax (50 µg neoantigen + 3.2 µg CpG), or C‐N‐LNPs‐vax^D18^ (50 µg neoantigen and 3.2 µg CHO‐CpG), SM102 or MC3‐based nanovaccines, with five to six mice per group. Mix‐vax, C‐N‐LNPs‐vax^D18^, SM102, or MC3‐based nanovaccines were administered intravenously to mice every 4 days for a total of three injections. Bioluminescence images were acquired using the IVIS Lumina X5 and analyzed for total radiance (photons per second) following luciferin injection (20 mg mL^−1^, 150 µL per mouse). After 14 days (*n* = 3 mice per group), the treated mice were euthanized, and their main organs were collected and fixed in 10% paraformaldehyde solution. The main organs were sectioned, stained with H&E, and examined under a microscope.

To confirm the critical role of CD4^+^ and CD8^+^ T cells in the C‐N‐LNPs‐vax^D18^ treatment, cell depletion studies of CD4^+^ and CD8^+^ T cells were performed using anti‐CD4 (100 µg, BioXCell) and anti‐CD8 (100 µg, BioXCell) depleting antibodies (Figure S23a, Supporting Information). Mice received intraperitoneal injections of anti‐CD4 or anti‐CD8 antibodies (day ‐17). Ten days later (day ‐7), Hepa1‐6‐Luc cells (3.5 × 10⁵) were injected under the liver capsule. Starting on day 1, the mice were intravenously administered PBS or C‐N‐LNPs‐vax^D18^ every 4 days for a total of three doses (days 1, 5, and 9). During nanovaccine administration, the mice continued to receive anti‐CD4 or anti‐CD8 antibodies intraperitoneally on days 0 and 7. Bioluminescence images were acquired using the IVIS Lumina X5 and analyzed for total radiance (photons per second) following luciferin injection (20 mg mL^−1^, 150 µL per mouse).

To evaluate T‐cell activation, T cells were obtained from the spleens of female C57BL/6 mice. The spleen was harvested and stored on ice. The cells were flushed out from the spleens using a medium and filtered through a 40 µm strainer to eliminate debris. After centrifugation, cells were resuspended in red blood cell lysis buffer and incubated for 10 min to lyse the cells. They were then centrifuged again and resuspended in a medium. Corresponding antibodies were used to stain the cells, which were then examined by FCM. The antibodies used for FCM included anti‐CD3‐APC (eBioscience), anti‐CD4‐FITC (eBioscience), and anti‐CD8‐PE (eBioscience) for TILs, and anti‐CD25‐PerCP‐Cy5.5 (eBioscience) and anti‐Foxp3‐PE‐Cy7 (eBioscience) for Tregs. To isolate and analyze memory T cells, the spleens were dissected and then filtered through a 40 µm filter by using Ficoll‐Paque density gradient centrifugation. The isolated cells were stained with the following antibodies (anti‐CD3‐APC (eBioscience, USA), anti‐CD4‐FITC (eBioscience, USA), anti‐CD8‐PE (eBioscience, USA), anti‐CD44‐PE‐Cy7 (eBioscience, USA), and anti‐CD62L‐PerCP‐Cy5.5 (eBioscience, USA)) and then examined using FCM.

To investigate the lymphocyte populations in the tumors and spleens after treatment, these tissues were extracted from orthotopic HCC mice. Primary tumors were digested with enzymes containing 1 mg mL^−1^ collagenase type IV, 0.2 mg mL^−1^ hyaluronidase, and 0.02 mg mL^−1^ DNase I to produce a single‐cell suspension. The resulting cells were subsequently stained with antibodies to evaluate the infiltrating T cell and Treg populations by FCM analysis. Specifically, T cells were stained with anti‐CD4‐FITC (eBioscienceTM, 11‐0042‐85), anti‐CD8‐PE (eBioscienceTM, 12‐0081‐82), anti‐CD3‐APC (eBioscienceTM, 17‐0032‐82), anti‐IFN‐γ‐PE‐Cy7 (eBioscienceTM, 11‐0042‐85), anti‐CD62L‐PerCP‐Cy5.5, and anti‐CD44‐PE‐Cy7 antibodies. For Treg analysis, the cell suspension was stained with anti‐CD4‐FITC, anti‐CD25‐PerCP‐Cy5.5 (eBioscienceTM 45‐0251‐82), and anti‐Foxp3‐PE‐Cy7 (eBioscienceTM, 25‐5773‐82) antibodies. For macrophage analysis, the cell suspension was also stained with anti‐CD11b‐APC, anti‐CD86‐PerCP‐Cy5.5 (eBioscienceTM 45‐0251‐82), anti‐CD80‐PE (eBioscienceTM, 25‐5773‐82), and anti‐CD206‐PE (eBioscienceTM 45‐0251‐82) antibodies. To evaluate any pathological changes in the tumors after treatment, tumor sections were stained with H&E for analysis.

### Antitumor Effect of C‐N‐LNPs‐vax^D18^ Combined with αPD1 Therapy In Vivo

To evaluate the antitumor efficacy of the combined treatment involving anti‐PD1 (αPD1) antibody and C‐N‐LNPs‐vax^D18^ (50 µg neoantigen and 3.2 µg CHO‐CpG) in the orthotopic Hepa1‐6 liver tumors or MC38 tumor mouse models, female C57BL/6 mice (aged 4–5 weeks) were initially inoculated with Hepa1‐6‐Luc cells (3.5 × 10^5^) directly under the liver capsule. After 7 days, the successful establishment of orthotopic Hepa1‐6 liver tumors was confirmed in mice using the IVIS Lumina X5 imaging system. MC38 cells (3 × 10^5^) were combined with a Matrigel suspension (25 µL) and injected into the flanks of female C57BL/6 mice (aged 4–5 weeks). Following a 7‐day incubation period post‐injection, C57BL/6 mice with established tumors were treated with either C‐N‐LNPs‐vax^D18^ or C‐N^MC38^‐LNP‐vax^D18^ via intravenous (i.v.) injection on days 0, 4, and 8, and received αPD1 antibody (3 mg kg^−1^) by intraperitoneal injection. Subsequently, bioluminescence images of Hepa1‐6 tumors were captured using the IVIS Lumina X5 and analyzed for total radiance (photons per second) after luciferin injection (20 mg mL^−1^, 150 µL per mouse). MC38 tumors were measured using a Vernier caliper every 3–4 days. Tumor volume was calculated using the following equation: *V*_tumor_ = (*A* × *B* ^2^) 1/2, where *A* and *B* are the longer and shorter diameters (mm) of the tumor, respectively.

### Statistical Analysis

Statistical analyses were conducted using one‐way analysis of variance (ANOVA) for comparisons between multiple groups, and/or the two‐tailed Student's *t*‐test was used for comparisons between two groups. GraphPad Prism software (version 8.0) was used for all analyses. Survival curves were constructed using Kaplan–Meier estimates and tested using the log‐rank test. The significance levels were set at ^*^p < 0.05 for statistical significance, ^**^p < 0.01, ^***^p < 0.001, ^****^p < 0.0001. *p *values* > *0.05 were considered not significant (ns). Pearson's correlation coefficient was used to evaluate the correlation matrices. All data are presented as means ± standard deviation (SD) (*n* ≥ 3).

## Conflict of Interest

The authors declare no conflict of interest.

## Supporting information



Supporting Information

## Data Availability

The data that support the findings of this study are available from the corresponding author upon reasonable request.
