# Supplementary material for: Targeting Antigen‐Presenting Cells to Enhance the Tumor‐Spleen Immunity Cycle through Liposome‐Neoantigen Vaccine
Source: Adv Sci (Weinh). 2025 Mar 24;12(19):2500021. doi: 10.1002/advs.202500021 (PMC12097013; doi:10.1002/advs.202500021)
Supplement: Supplementary file 1 — Supporting Information [file ADVS-12-2500021-s001.docx]

**Supporting Information**

**Targeting Antigen-Presenting Cells to Enhance the Tumor-Spleen Immunity Cycle through Ligand-free Liposome-Neoantigen Vaccine**

Yu Xu^1,2^^#^, Bing Wang^5,8#^, Yue Huang^1,2#^, JianPing Liao^3#^, Chenyi Wu^1,2#^, Chenxi Zhou^5^, Zishi Kang^5^, Shiyang Jiang^5^, Bing-Chen Wu^5^, Da Zhang^1,2,5,7*^, Ruihua Xu^2,4*^, Xiaolong Liu^5,6,7*^, Feng Wang^1,2*^

Dr. Y. X, Y. H, J. L, C. W, Prof. D. Z, Prof. R. X, Prof. F. W,

^1^Department of Medical Oncology, Sun Yat-sen University Cancer Center, State Key Laboratory of Oncology in South China, Collaborative Innovation Center for Cancer Medicine, Sun Yat-sen University, Guangzhou 510060, P. R. China.

Dr. Y. X, Y. H, J. L, C. W, Prof. D. Z, Prof. R. X, Prof. F. W,

^2^Research Unit of Precision Diagnosis and Treatment for Gastrointestinal Cancer, Chinese Academy of Medical Sciences, Guangzhou 510060, P. R. China.

Dr. J. L,

^3^Sun Yat-sen University Cancer Center, State Key Laboratory of Oncology in South China, Collaborative Innovation Center for Cancer Medicine, Sun Yat-sen University, Guangzhou 510060, P. R. China.

Prof. R. X,

^4^Department of Medical Oncology, Sun Yat-sen University Cancer Center, State Key Laboratory of Oncology in South China, Guangdong Provincial Clinical Research Center for Cancer, Sun Yat-sen University, Guangzhou 510060, P. R. China.

B. W, C. Z, Z. K, S. J, B. W, Prof. D. Z, Prof. X. L,

^5^The United Innovation of Mengchao Hepatobiliary Technology Key Laboratory of Fujian Province, Mengchao Hepatobiliary Hospital of Fujian Medical University, Fuzhou 350025, P. R. China.

Prof. X. L,

^6^CAS Key Laboratory of Design and Assembly of Functional Nanostructures, Fujian Institute of Research on the Structure of Matter, Chinese Academy of Sciences, Fuzhou 350002, P. R. China.

Prof. D. Z, Prof. X. L,

^7^Mengchao Med-X Center, Fuzhou University, Fuzhou 350116, P. R. China.

B. W,

^8^Fujian Agriculture and Forestry University, Fuzhou 350002, P. R. China.

*E-mail: [zdluoman1987@163.com](mailto:zdluoman1987@163.com), [xurh@sysucc.org.cn](mailto:xurh@sysucc.org.cn), [xiaoloong.liu@gmail.com](mailto:xiaoloong.liu@gmail.com), [wangfeng@sysucc.org.cn](mailto:wangfeng@sysucc.org.cn)

^#^These authors contributed equally to this work.

**Table S1.** The summarizing of different C-LNPs-vax formulations.

|  | **DOTMA（μM）** | **DOPE（μM）** | **CHO（μM）** | **DDBA（μM）** | **Neoantigen（mg）** | **CHO-CpG（nM）** |
| --- | --- | --- | --- | --- | --- | --- |
| **C-LNPs-vax^D18^** | 1 | 1 | 0.25 | 0.5 | 1 | 10 |
| **C-LNPs-vax^D30^** | 1 | 1 | 0.25 | 1 | 1 | 10 |
| **C-LNPs-vax^D47^** | 1 | 1 | 0.25 | 2 | 1 | 10 |
| **C-LNPs-vax^D64^** | 1 | 1 | 0.25 | 4 | 1 | 10 |
| **C-LNPs-vax^D72^** | 1 | 1 | 0.25 | 6 | 1 | 10 |
| **C-LNPs-vax^D78^** | 1 | 1 | 0.25 | 8 | 1 | 10 |


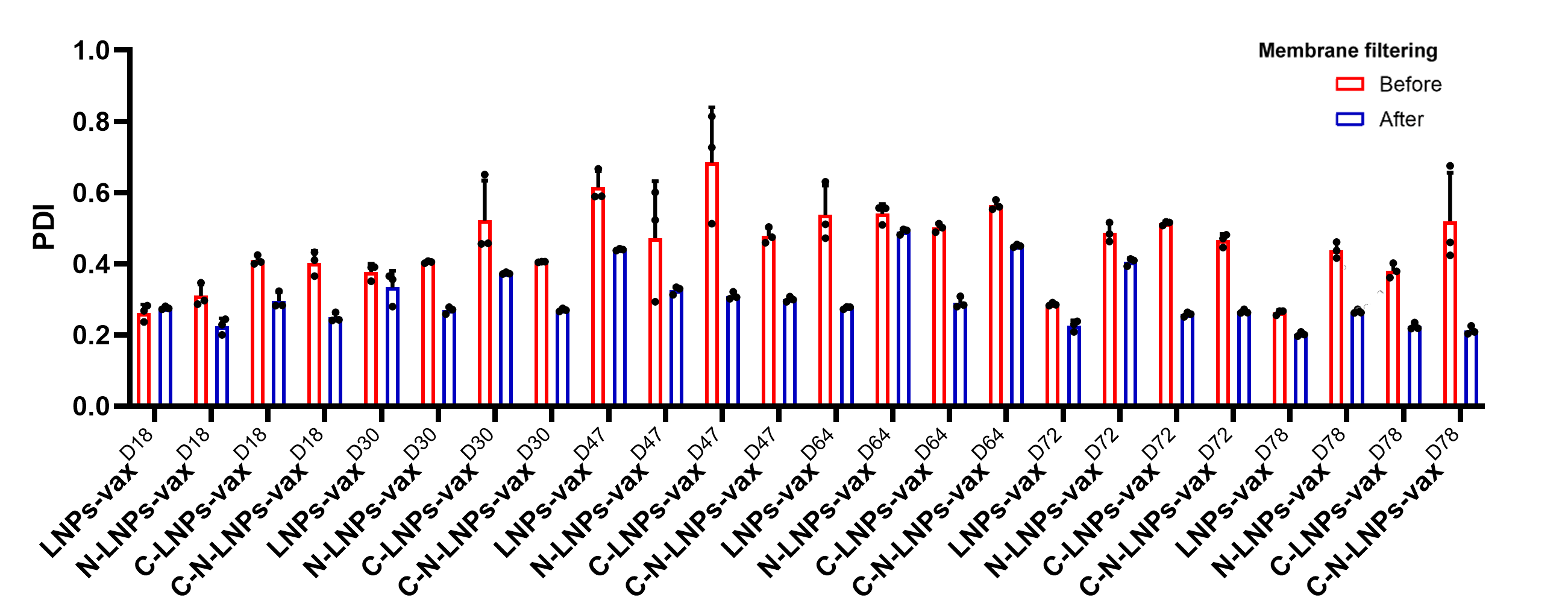


**Figure S1.** The PDI value of various formulations of LNPs, neoantigen loaded LNPs (N-LNPs), CHO-CpG loaded LNPs (C-LNPs) or C-N-LNPs-vax before and after extrusion through the filtration membrane, n = 3. Data are expressed as mean ± SD.


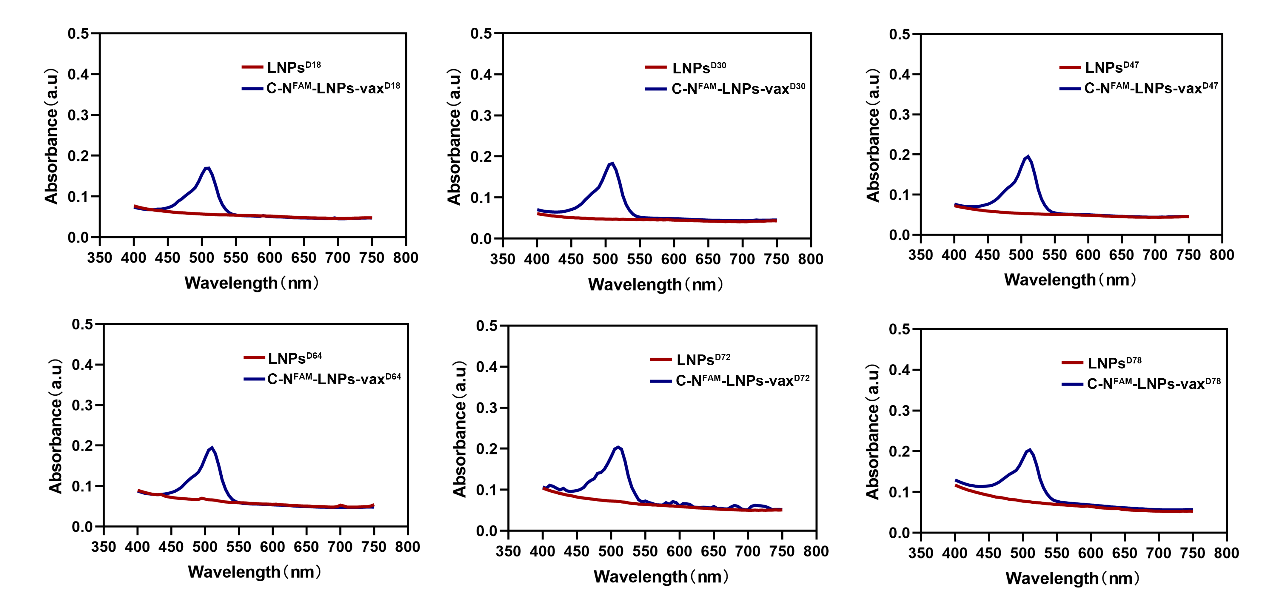


**Figure S2.** UV-vis spectra of various formulations of LNPs and C-N^FAM^-LNPs-vax (^FAM^Neoantigen) to affirm the successful loading of Neo-antigens.


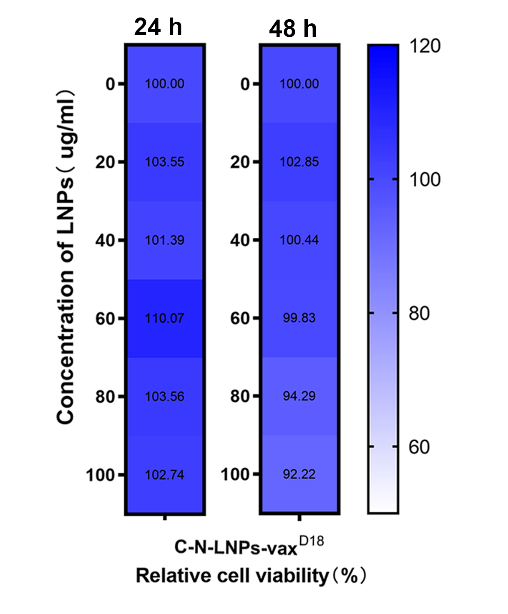


**Figure S3.** Cell viability of DCs.2.4 cells after co-incubation with different concentrations of C-N-LNPs-vax^D18^ for 24hrs and 48hrs, respectively, (n = 5). Data are expressed as mean ± SD.


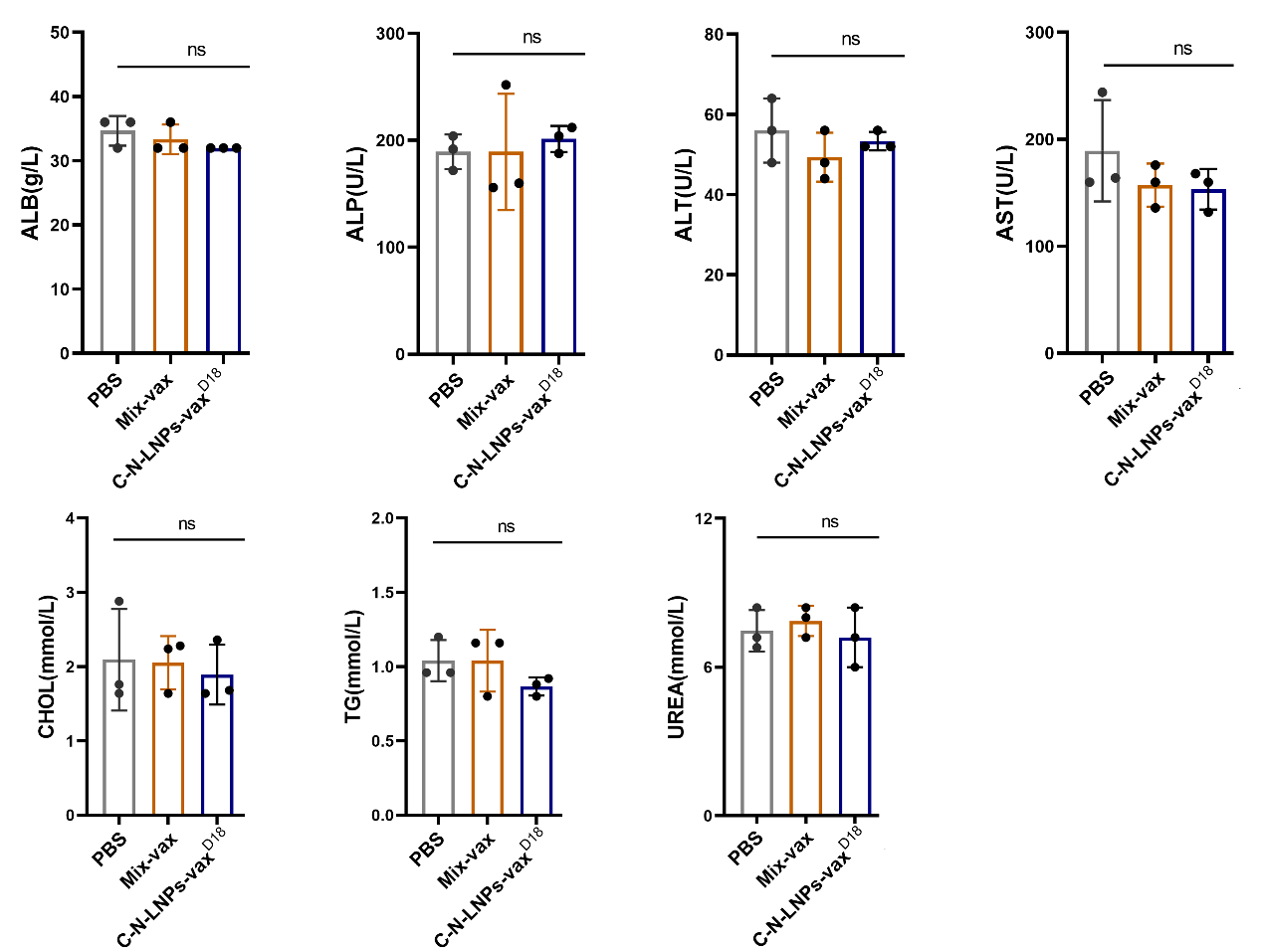


**Figure S4.** Blood biochemical analysis (ALB, ALP, ALT, AST, CHOL, TG and UREA) after *i.v.* injection of PBS, Mix-vax, or C-N-LNPs-vax^D18^ into C57BL/6 female mice with therapeutic dose (n = 3). Statistical analysis was performed with ANOVA analysis, *P values > 0.05 were considered not significant (ns).* Data are presented as mean ± SD.


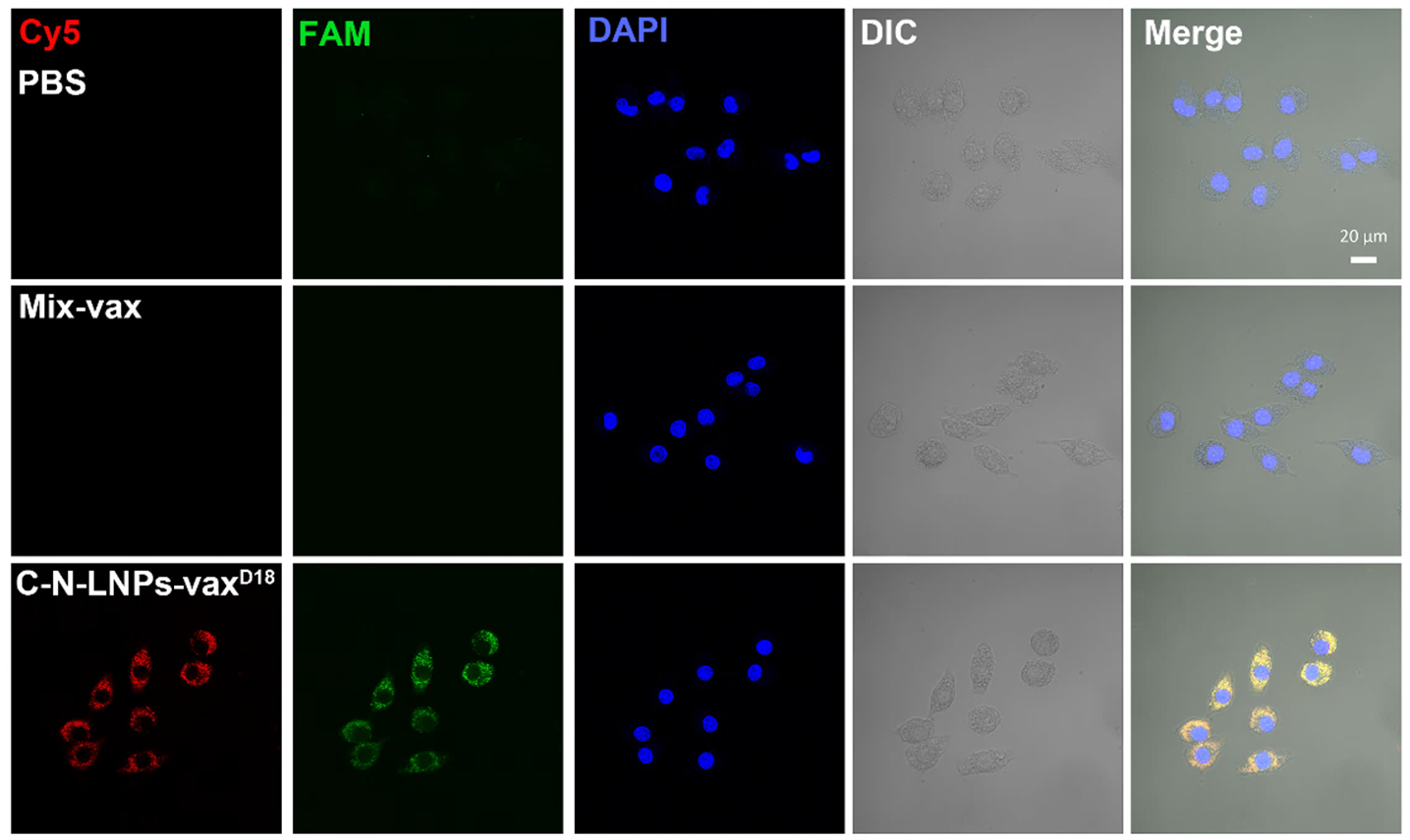


**Figure S5.** DCs2.4 cells treated with PBS, Mix-vax (CpG^Cy5^ and ^FAM^NeoAgs) or ^Cy5^C-^FAM^N-LNPs-vax^D18^ after 12hrs co-incubation, respectively. The cell nucleus was stained by DAPI. Scale bar, 20 μm.


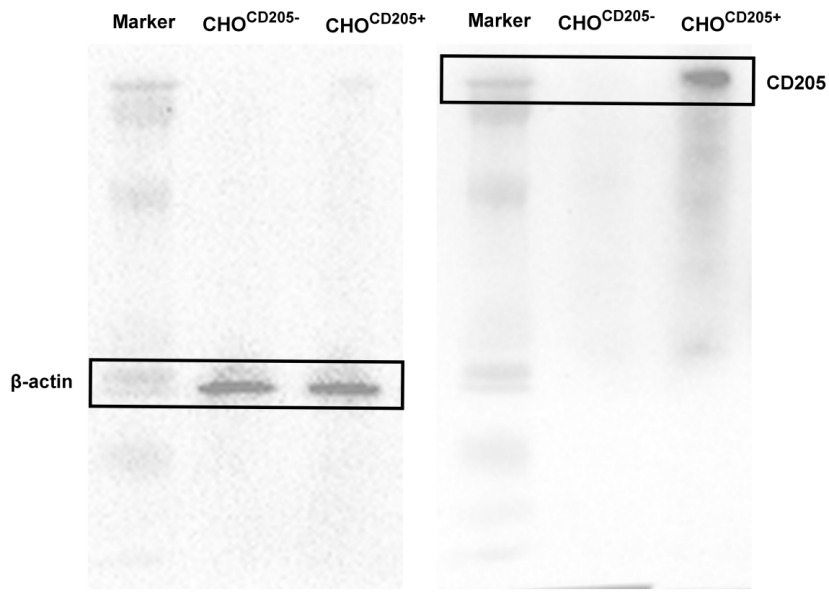


**Figure S6.** Western blotting analysis of CD205 in Chinese hamster ovary (CHO) cells that over-expressed mouse CD205 after transfection.


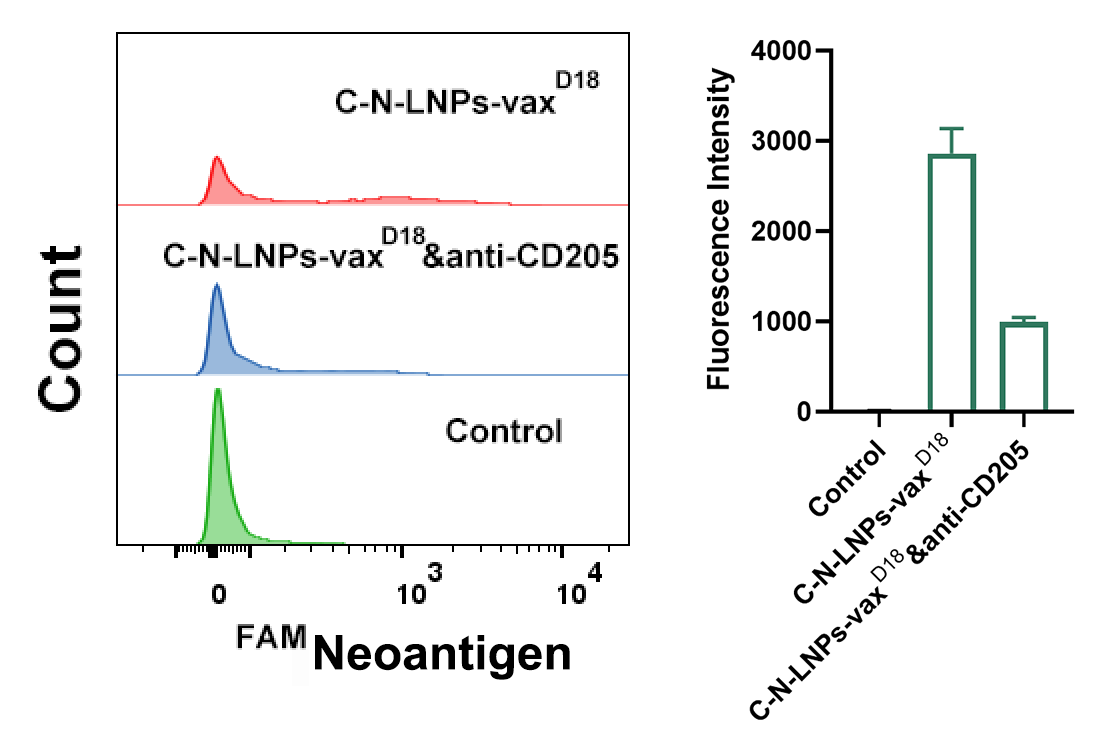


**Figure S7.** BMDCs were treated with PBS, or anti-CD205 antibodies for 12hrs, and then co-incubated with C-^FAM^N-LNPs-vax^D18^ for 12hrs co-incubations, respectively. The treated cells were then analyzed by FCM, n = 3. Data are expressed as mean ± SD.


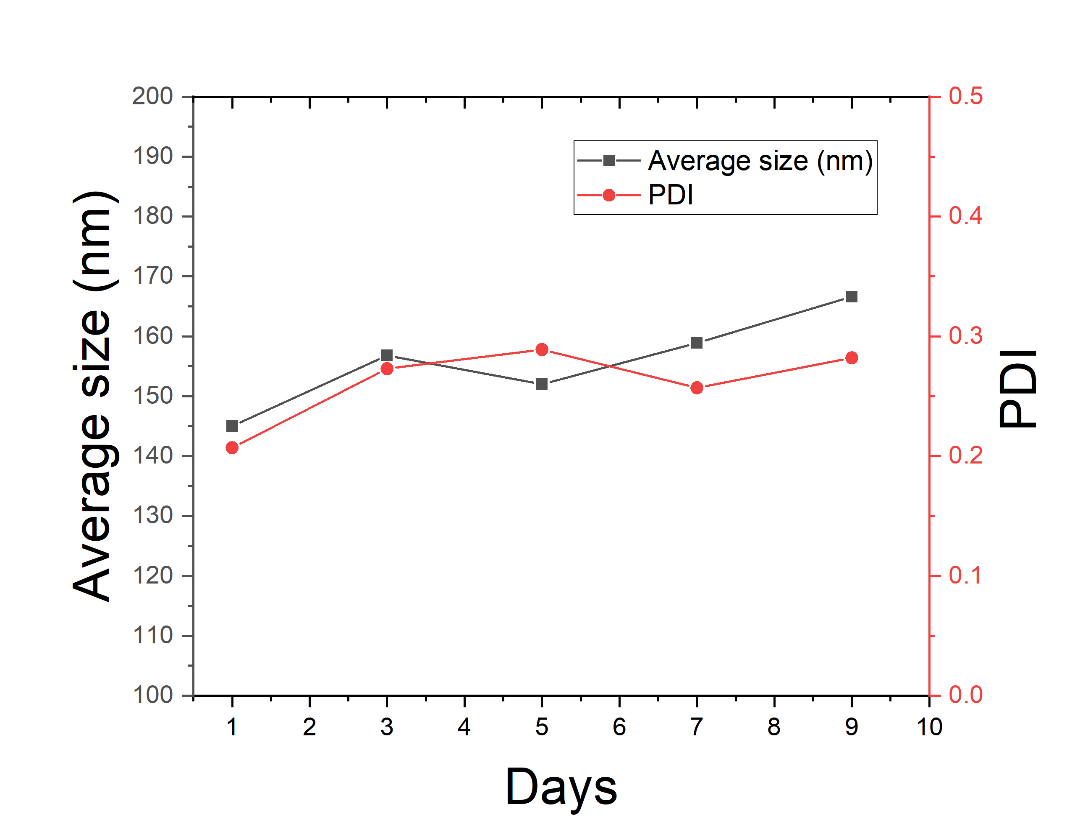


**Figure S8.** The DLS size and PDI value change of C-N-LNPs-vax^D18^ solution that contained 10% fetal bovine serum (FBS) during 10 days monitoring.


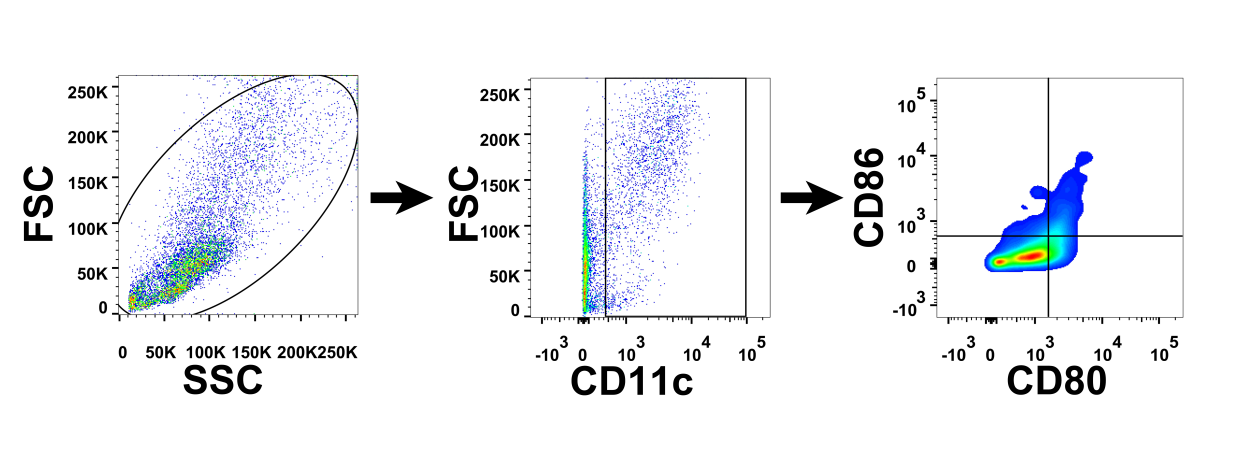


**Figure S9.** Representative gating strategy for BMDCs maturation analysis in Fig. 2I. Mature DC cells were defined as CD11c^+^-APC/CD80^+^-PE/CD86^+^-PE-Cy7.


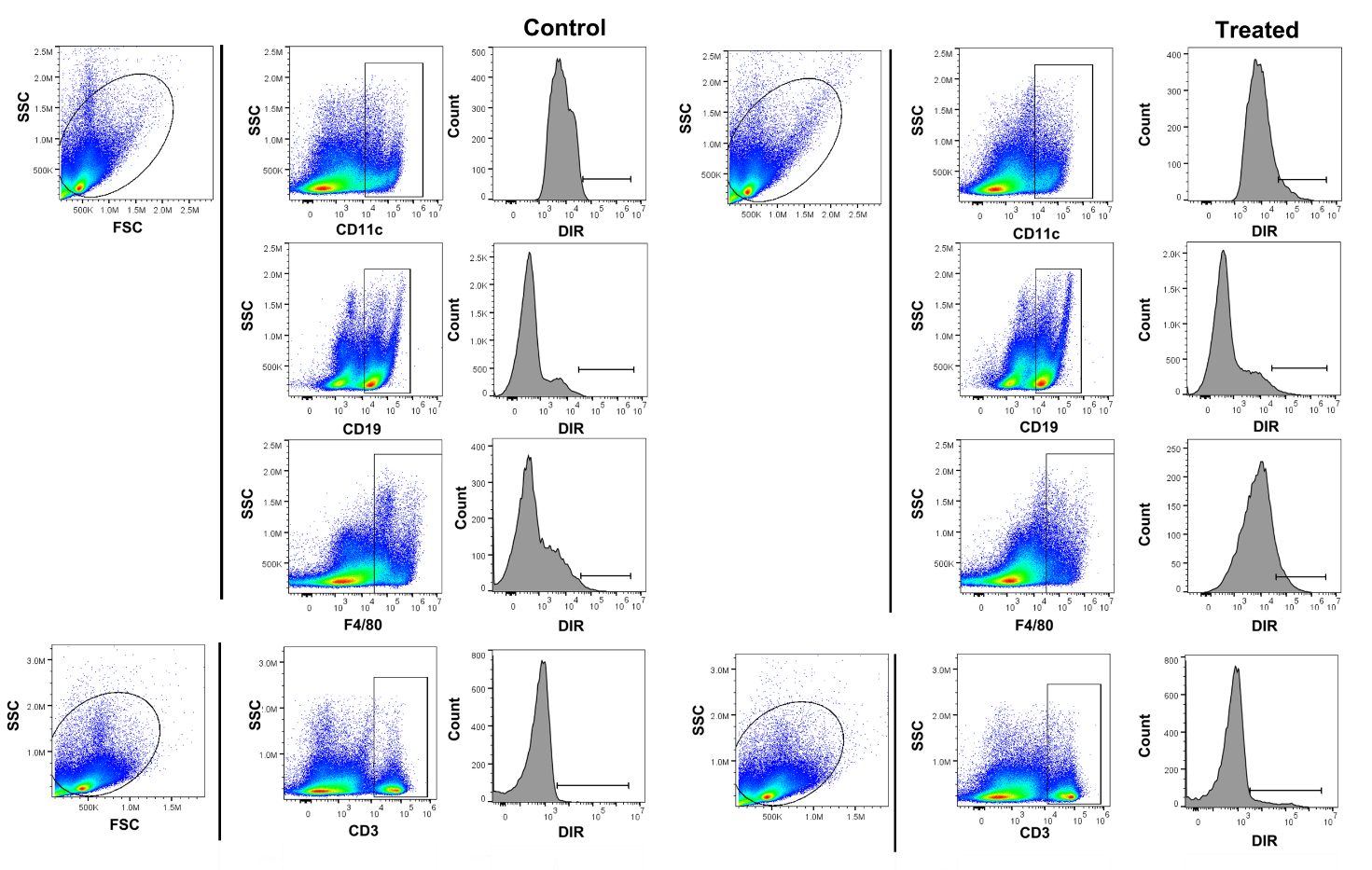


**Figure S10.** The gating strategy of different immune cells from the spleen analyzed by FCM with specific-biomarker staining as indicated.


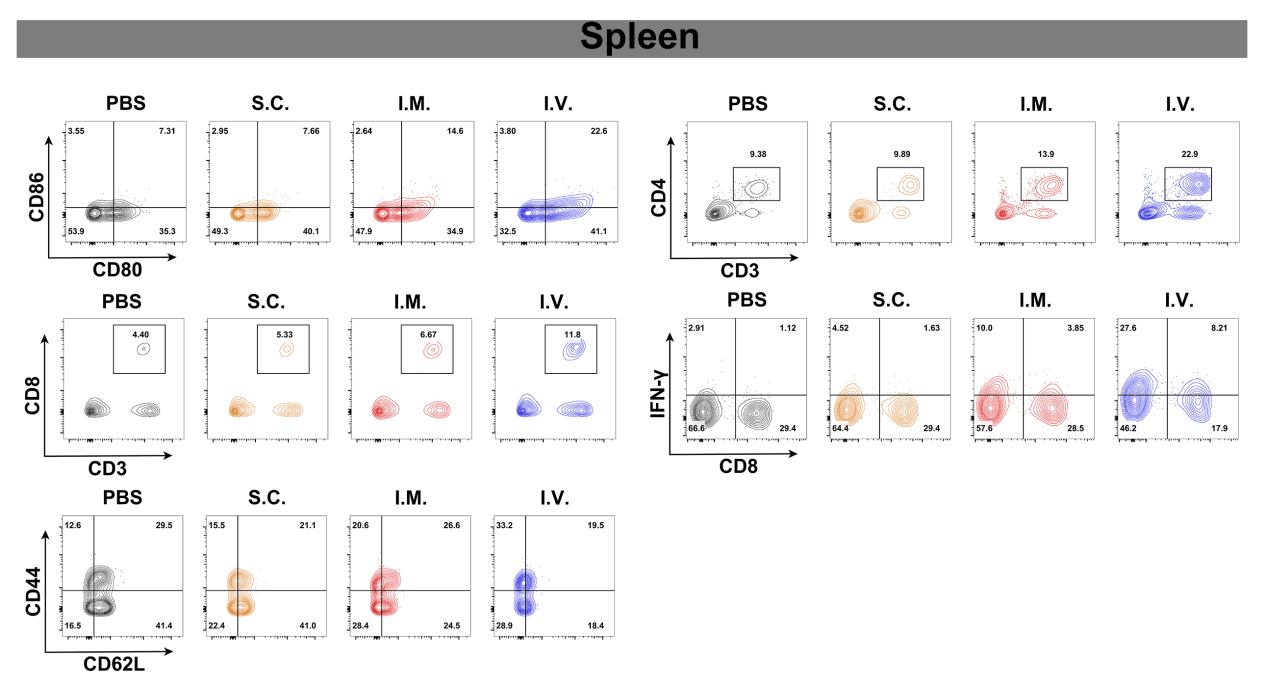


**Figure S11.** Representative gating strategy for antitumor immune response in Fig. 4B-4F. The mature DC cells were defined as CD11c^+^-APC/CD80^+^-PE/CD86^+^-PE-Cy7. T helper cells were defined as CD3^+^-APC / CD4^+^-FITC. Cytotoxic T cells were defined as CD3^+^-APC/CD8^+^-PE. IFN-γ^+^ producting T cells (Tc1) were defined as CD3^+^-APC/CD8^+^- PE/IFN-γ^+^-PE-Cy7; Memory T cells cells were defined as CD3^+^- APC/CD8^+^-PE/CD44^+^-PE-Cy7/CD62L^+^-Percp-Cy5.5.


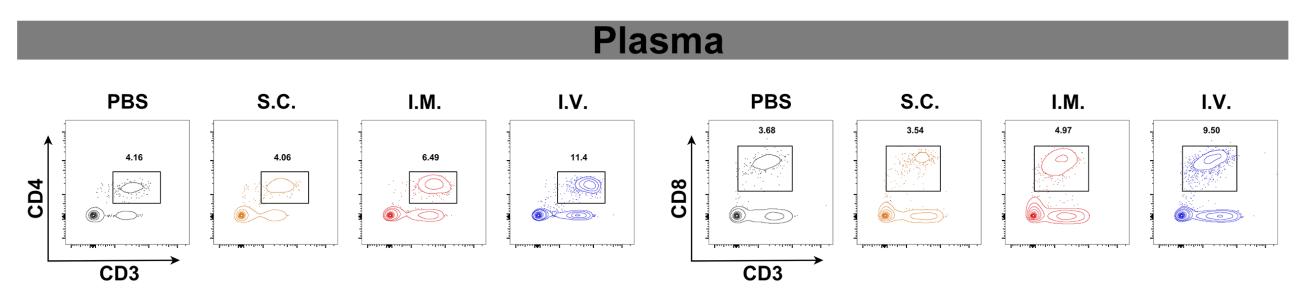


**Figure S12.** Representative gating strategy for antitumor immune response in Fig. 4G-4H. The T hepler cells were defined as CD3^+^-APC/CD4^+^-FITC; Cytotoxic T cells were defined as CD3^+^-APC/CD8^+^-PE.


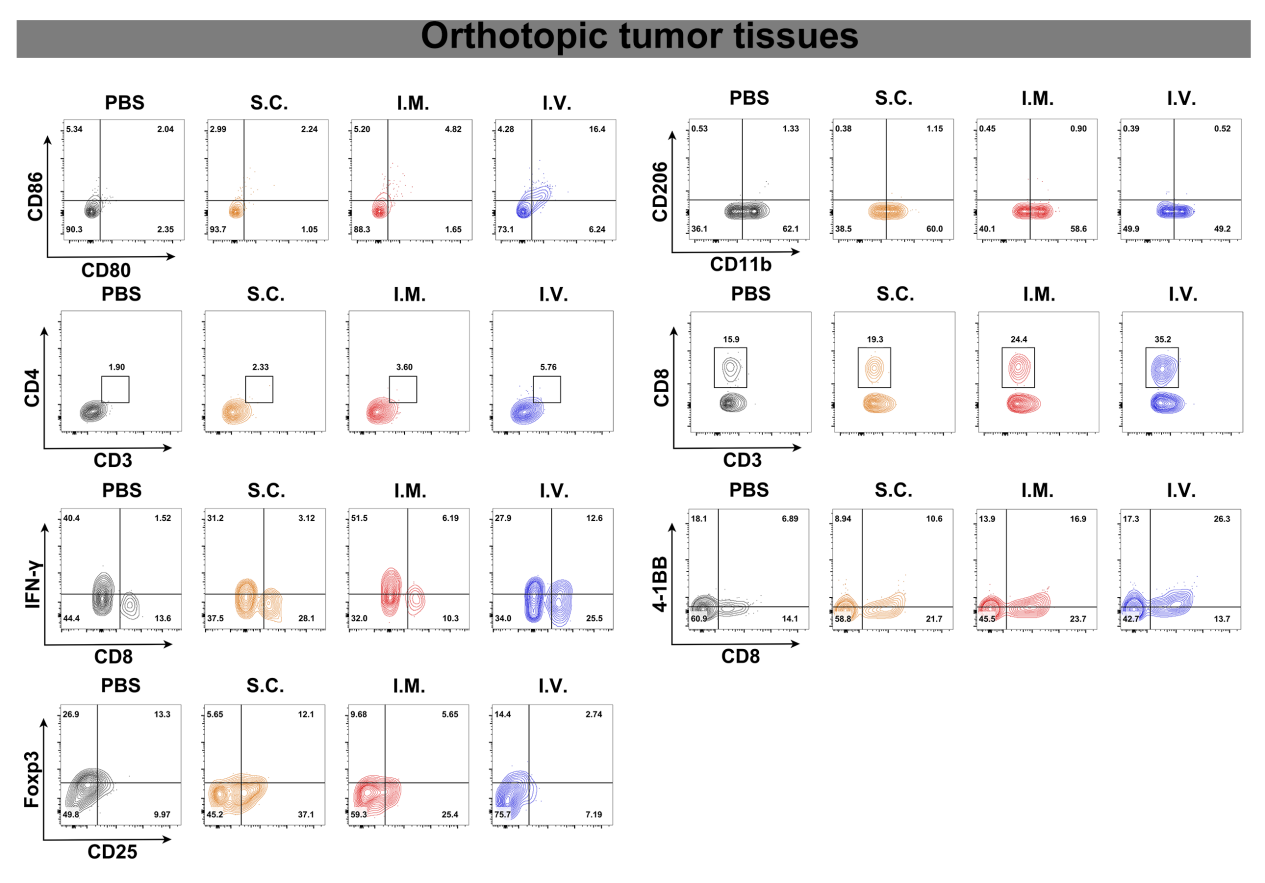


**Figure S13.** Representative gating strategy for antitumor immune response in Fig. 4I-4O. M1-TAMs were defined as CD11b^+^-APC/CD80^+^-PE/CD86^+^-PE-Cy7; M2-TAMs were defined as CD11b^+^APC/CD206^+^-PE; T hepler cells were defined as CD3^+^-APC/CD4^+^-FITC; Cytotoxic T cells were defined as CD3^+^-APC/CD8^+^-PE; IFN-γ^+^ producting CD8+T cells (Tc1) were defined as CD3^+^-APC/CD8^+^-PE/IFN-γ^+^-PE-Cy7; The activated CD8^+^T cells were defined as CD3^+^-APC/ CD8^+^-PE/4-1BB^+^-APC; The regulatory T cells (Tregs) were defined as CD4^+^-FITC/ CD25^+^ -Percp-Cy5.5 / Foxp3^+^-PE-Cy7.


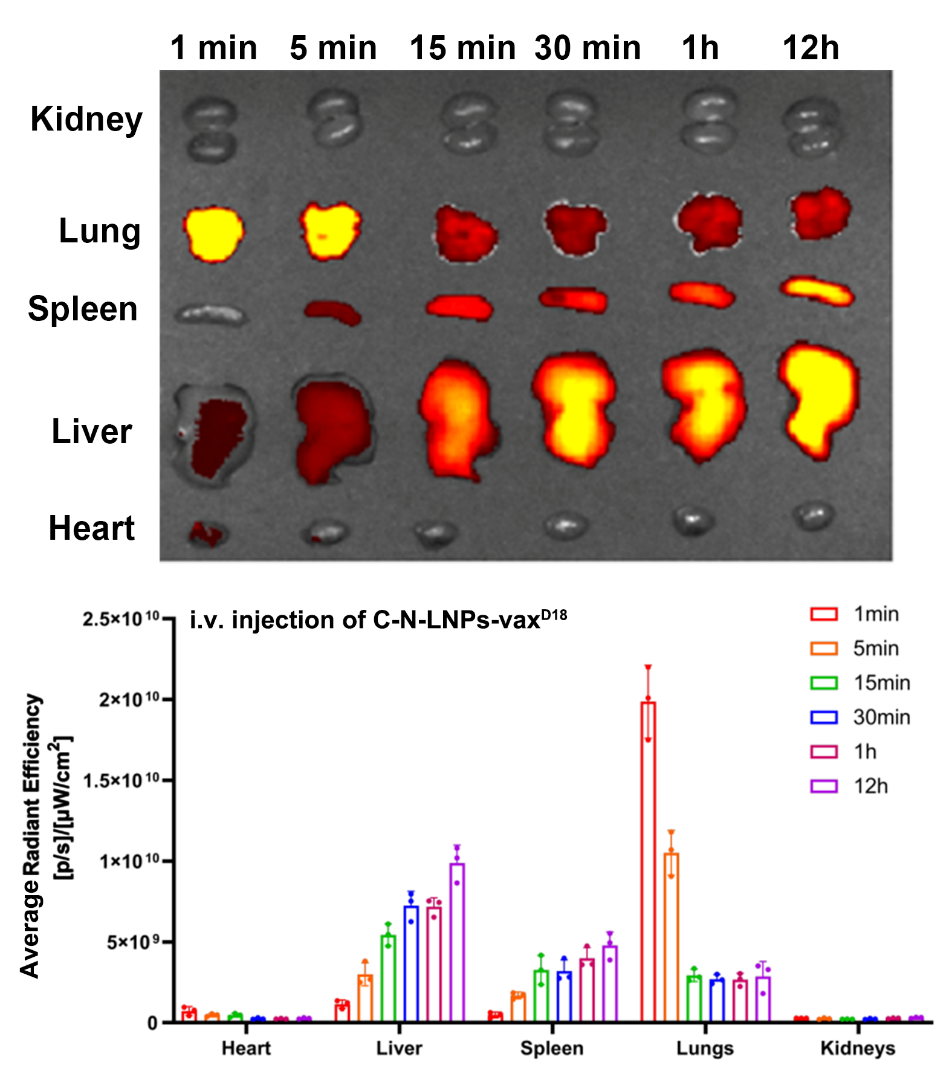


**Figure S14.** The bio-distribution of C-N-LNPs-vax^D18^ after intravenous (i.v.) injection for different time points, (n = 3). Data are expressed as mean ± SD.


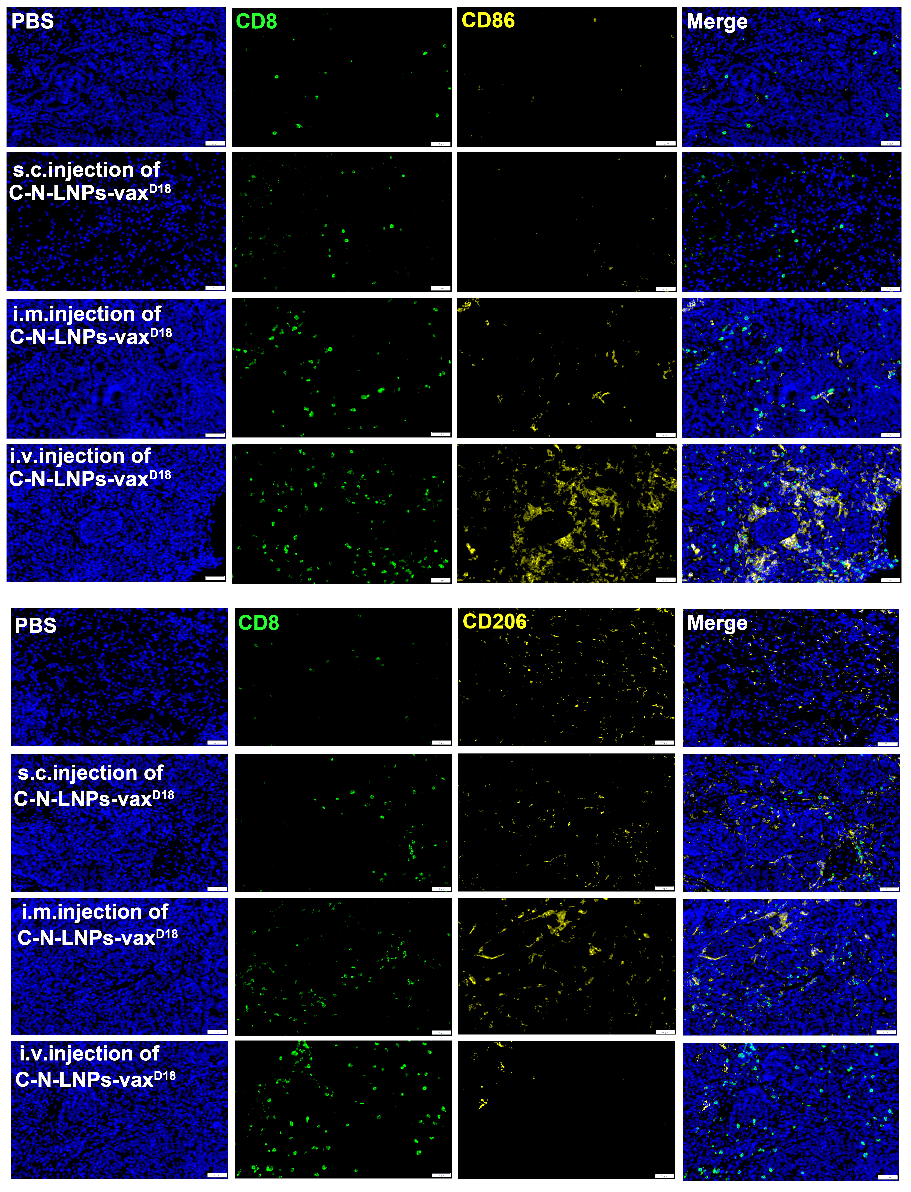


**Figure S15.** Multiplex immunoﬂuorescence staining for the CD8, CD86, and CD206 biomarkers for representing TILs, M1-TAMs, and M2-TAMs in Hepa1-6 tumors after different treatments as indicated.


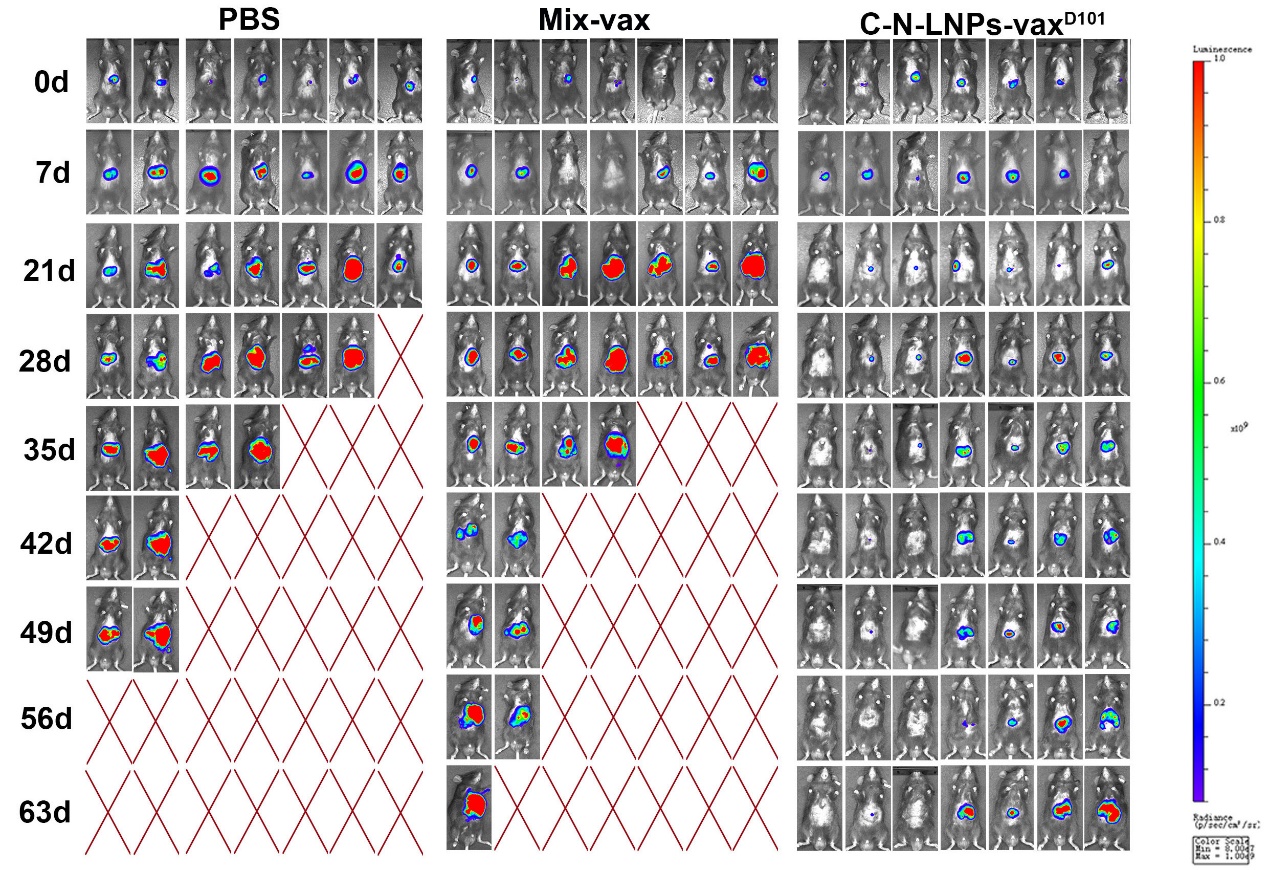


**Figure S16.** Bioluminescence imaging of Hepa1-6-luc-bearing mice every 7 days after intravenous injection of PBS, Mix-vax, or C-N-LNPs-vax^D18^, administered three times per four days, and the monitoring was performed until day 63, n = 7.


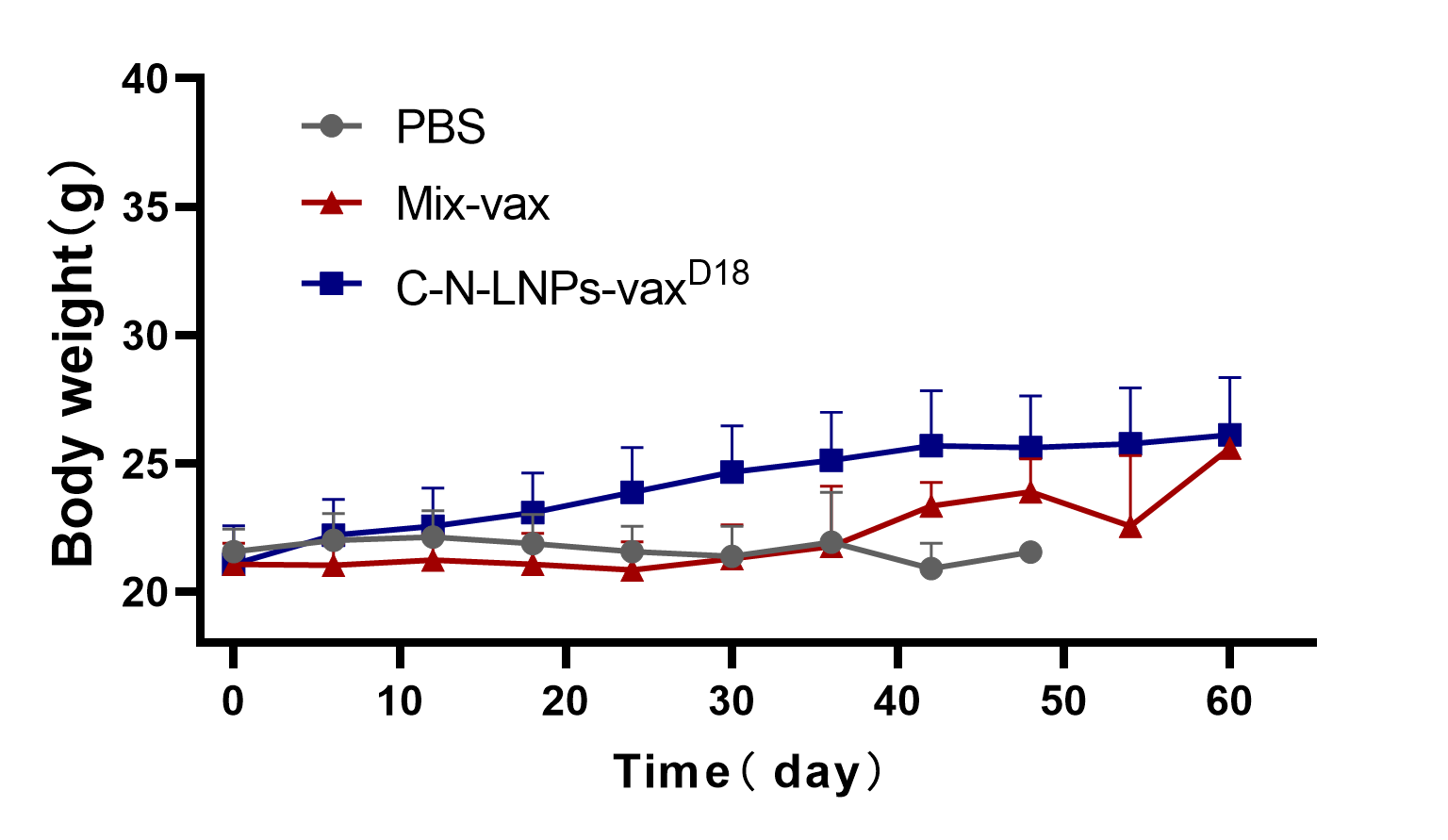


**Figure S17.** Body weight of mice after *i.v.* injection of PBS, Mix-vax, or C-N-LNPs- vax^D18^ for three times per four days, and the monitoring was performed until day 60, n = 7. Data are expressed as mean ± SD.

_
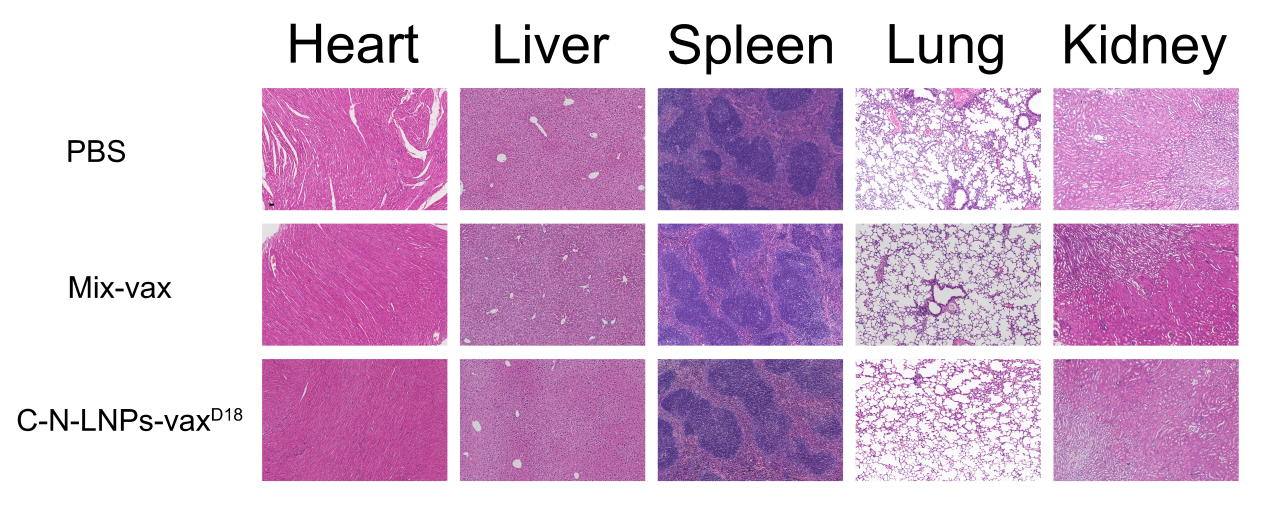
_

**Figure S18.** H&E staining of the major organs from C57BL/6 mice after *i. v.* injection of PBS, Mix-vax, or C-N-LNPs-vax^D18^ for 63 days later, Scale bar: 500 μm.


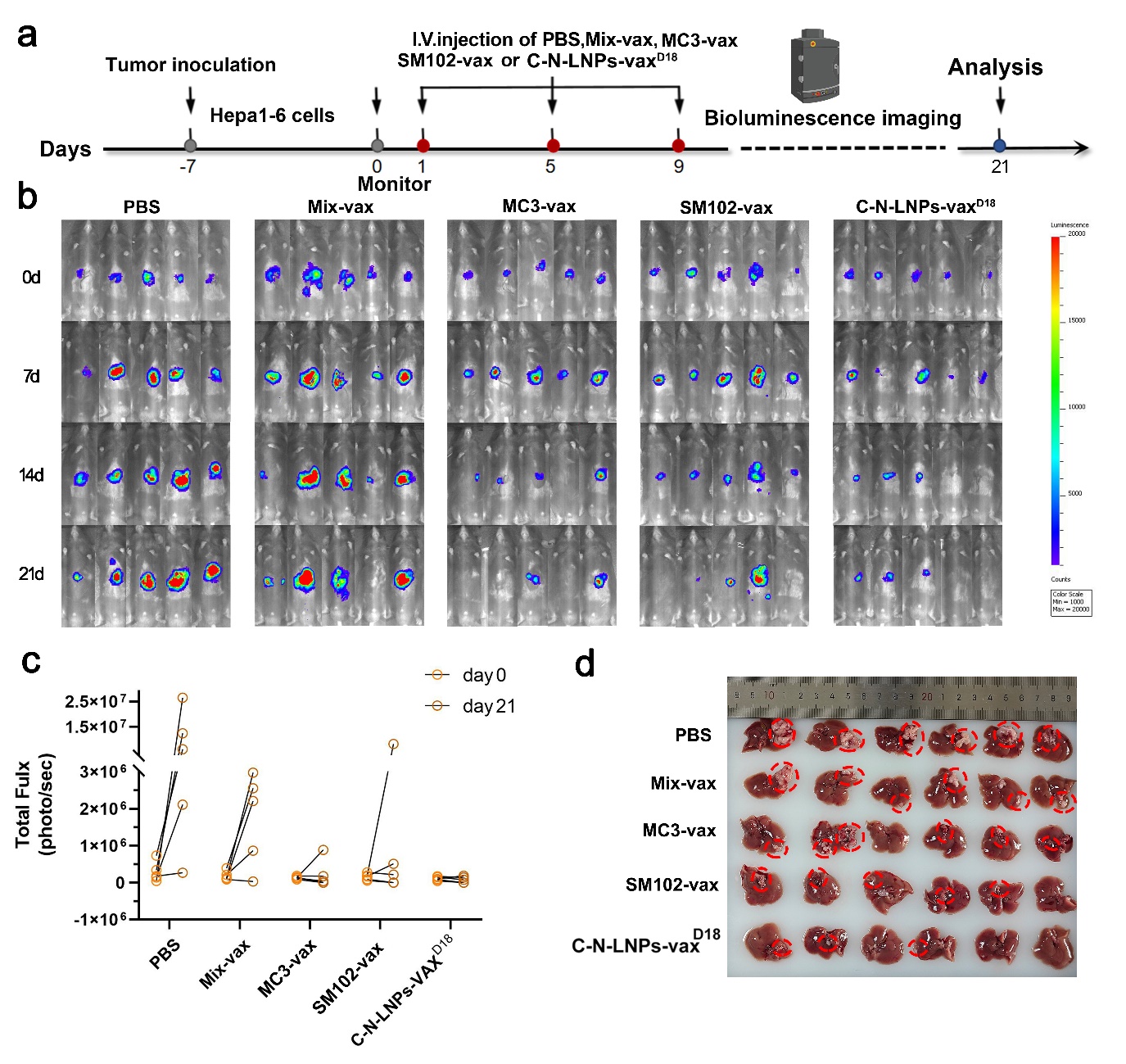


**Figure S19.** (A) Diagram of the treatment schedule and analysis of established orthotopic Hepa1-6-luc tumor models after i. v. injection of PBS, Mix-vax, MC3-based nanovaccine (MC3-vax), SM102-based nanovaccine (SM102-vax) or C-N-LNPs-vax^D18^ three times per 4 days. (B) Representative bioluminescence imaging on days 0, 7, 14, 21d post tumor inoculation (n = 5) after treatment as indicated in the established orthotopic Hepa1-6-luc bearing mice. (C) The total flux at 0 and 21 d before and after receiving different treatments as indicated (n = 5). Data are expressed as indiviual points. (D) The photo image of ex tumor and livers from orthotopic Hepa1-6-luc bearing mice after indicated treatment on day 21 (n = 6).


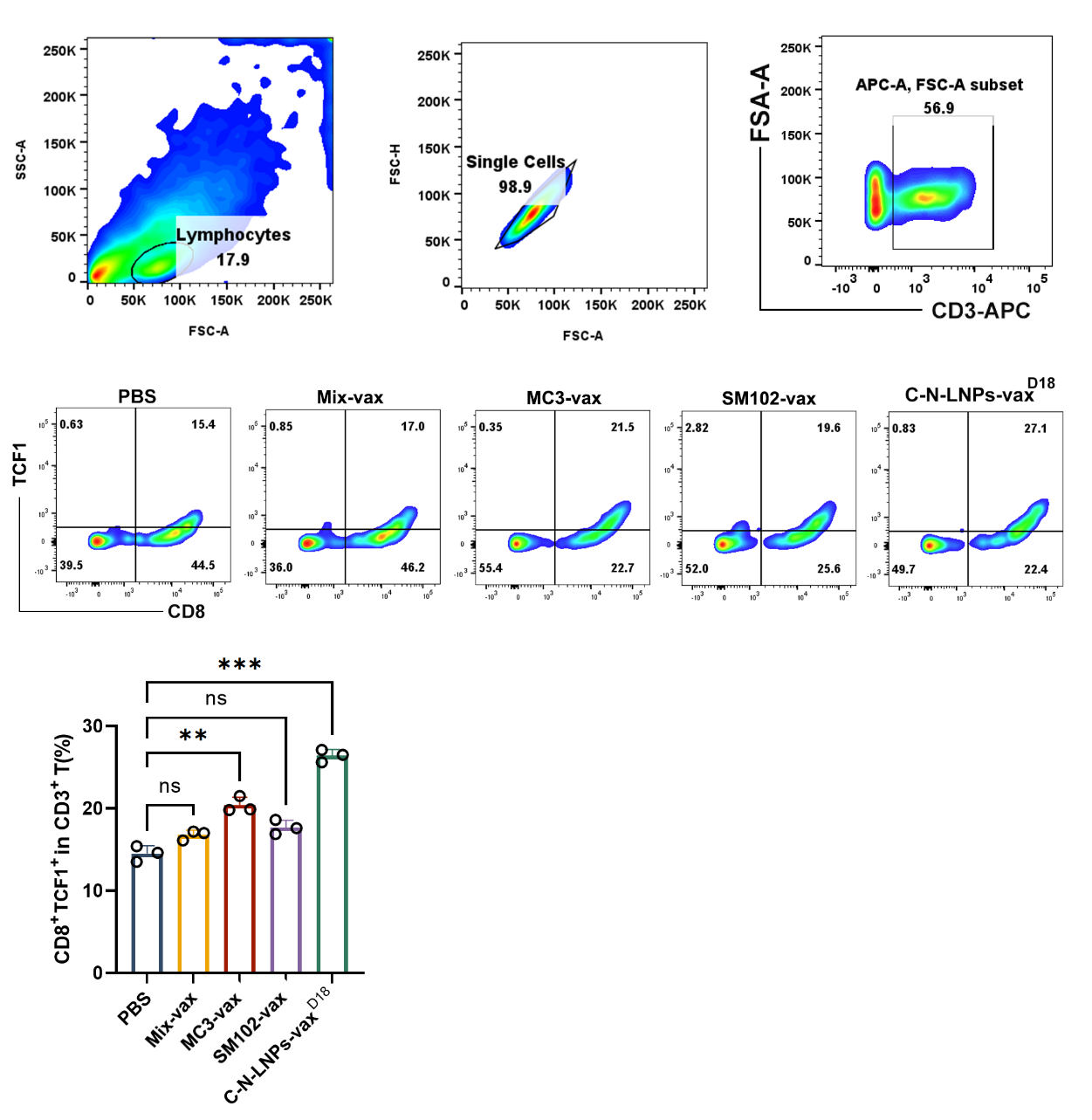


**Figure S20.** Representative gating strategy and TCF1^+^CD8^+^T cell populations in CD3^+^T cells of tumor tissues after received different treatment as indicated, n = 3. Statistical analysis was performed using ANOVA analysis, **p<0.05, **p<0.01, ***p<0.001, ****p<0.0001*. Data are expressed as mean ± SD.


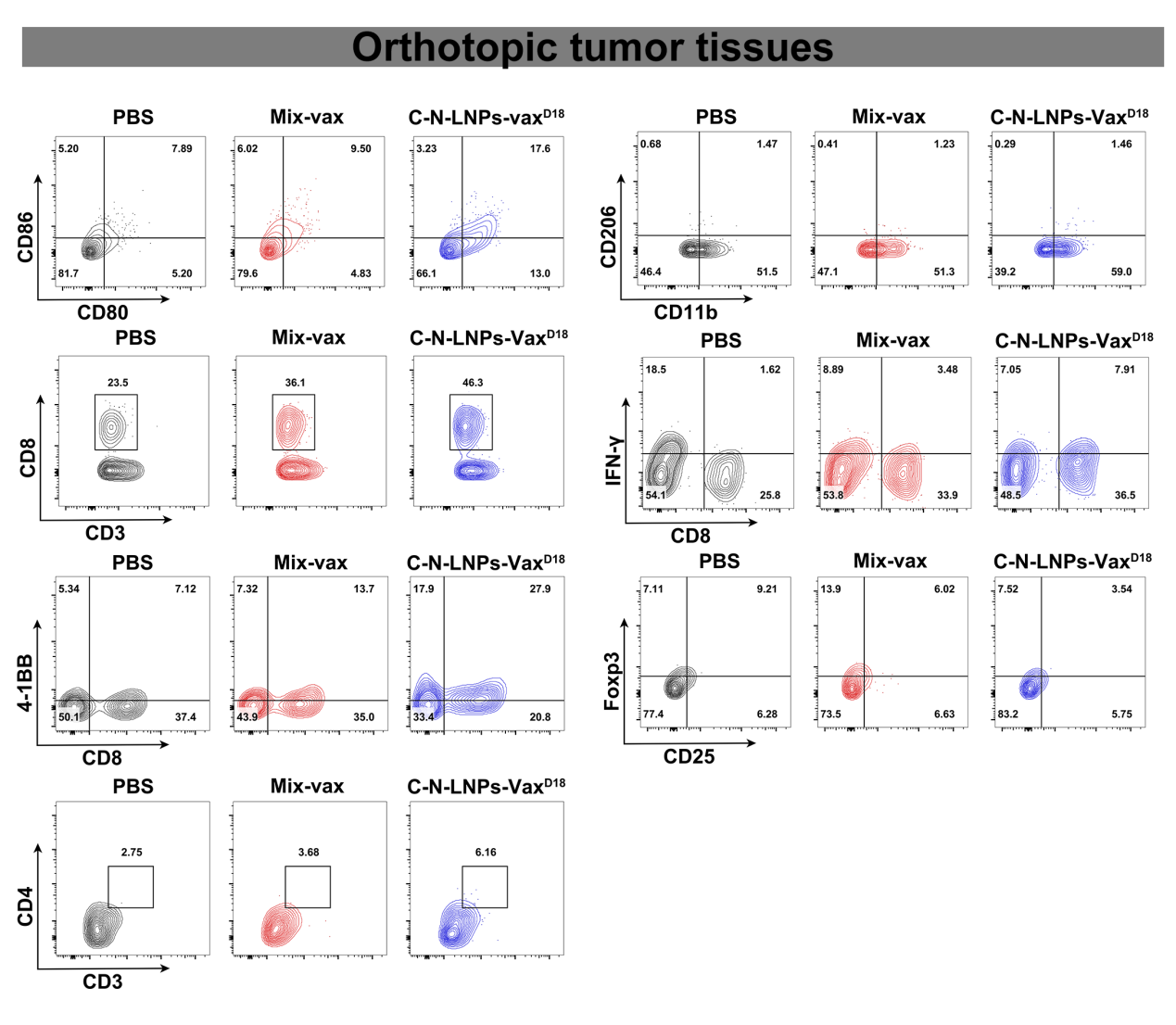


**Figure S21.** Representative gating strategy for antitumor immune responses in Fig. 6B-6H. M1-TAMs were defined as CD11b^+^-APC/CD80^+^-PE/CD86^+-^PE-Cy7; M2-TAMs were defined as CD11b^+^APC / CD206^+^PE; Cytotoxic T cells were defined as CD3^+^-APC/CD8+-PE; IFN-γ+producting CD8+T cells were defined as CD3+(APC) /CD8+(PE)/IFN-γ+(PE-Cy7); the activated T cells were defined as CD3^+^-APC/ CD8^+^-PE/4-1BB^+^-APC; the regulatory T (Tregs) cells were defined as CD4+-FITC / CD25^+^-Percp-Cy5.5/Foxp3^+^-PE-Cy7; T helper cells were defined as CD3^+^-APC / CD4^+^-FITC.


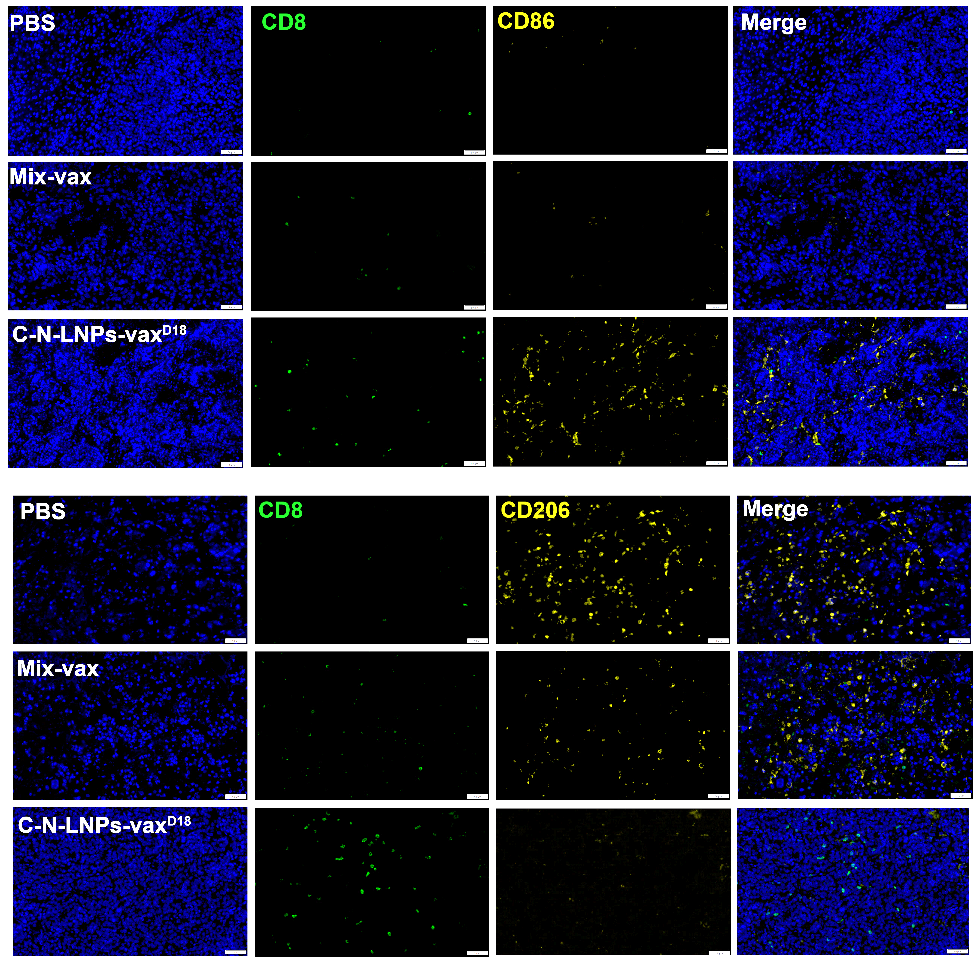


**Figure S22.** Multiplex immunoﬂuorescence staining for the CD8, CD86, and CD206 biomarkers for representing TILs, M1-TAMs, and M2-TAMs in Hepa1-6 tumors after different treatments as indicated.


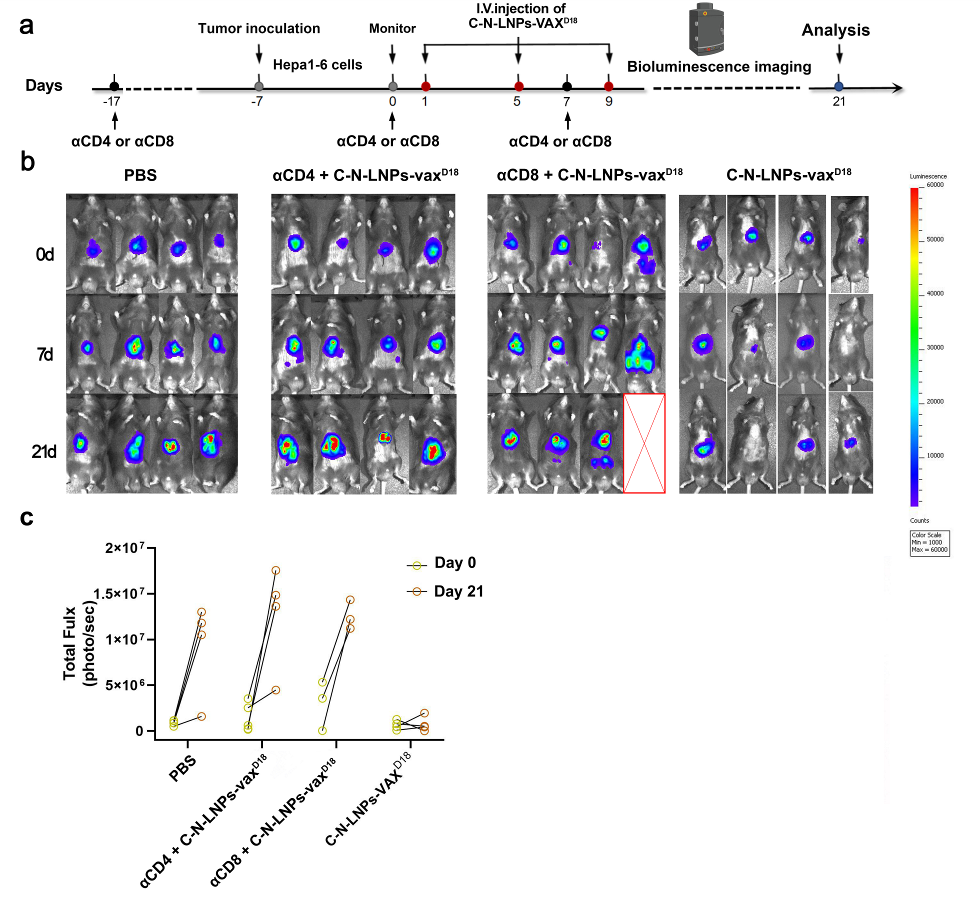


**Figure S23.** (A) Diagram of the treatment schedule and analysis of established orthotopic Hepa1-6-luc tumor models after i.v. injection of PBS, C-N-LNPs-vax^D18^ with or without anti-CD4 or anti-CD8 antibodies three times per 4 days. (B) Representative bioluminescence imaging on days 0, 7, 21 d post tumor inoculation (n = 4) after treatment as indicated in the established orthotopic Hepa1-6-luc bearing mice. (C) The total flux at 0 and 21 d before and after receiving different treatments as indicated (n = 3~4). Data are expressed as indiviual points.


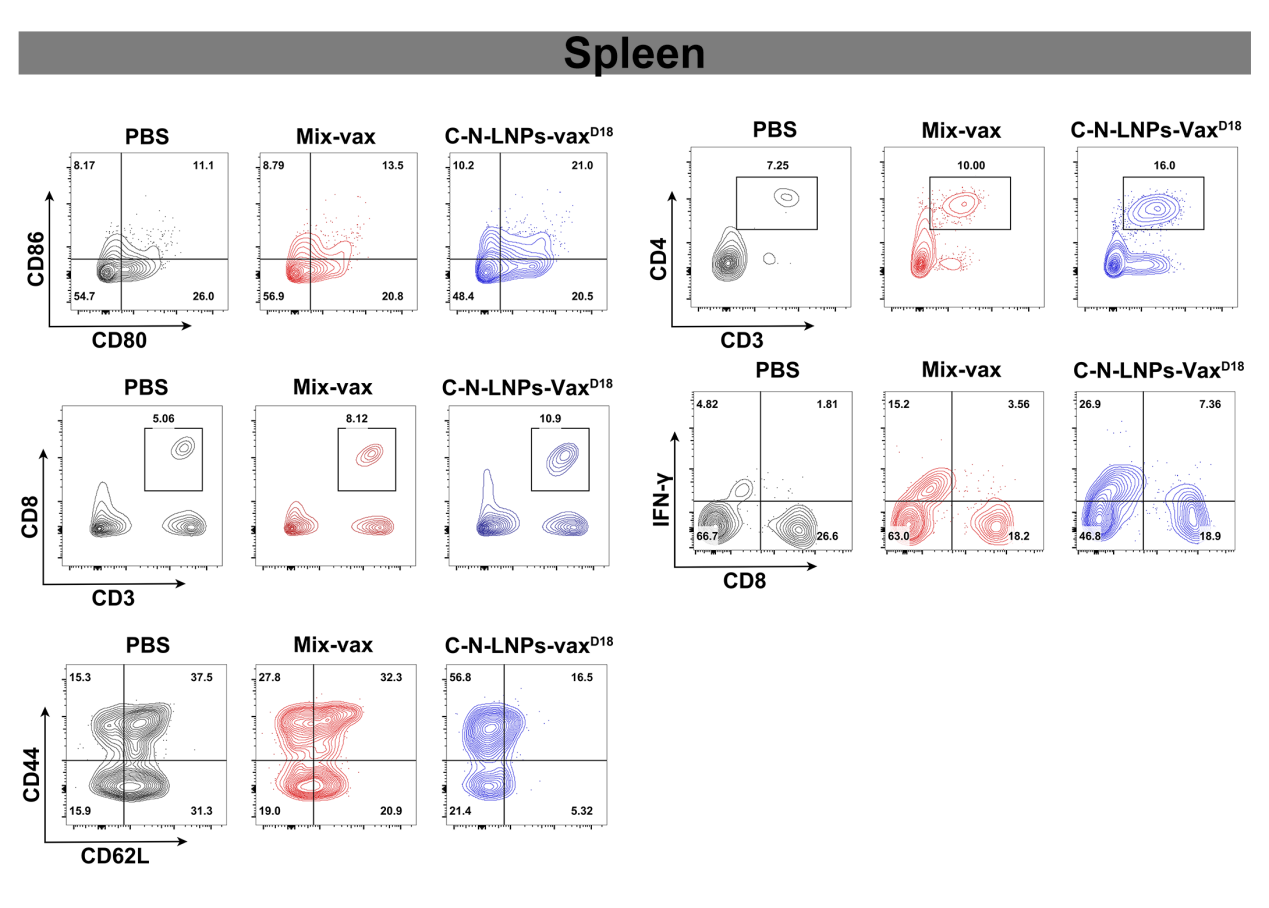


**Figure S24.** Representative gating strategy for antitumor immune response in Fig. 6J-6O. The mature DC cells were defined as CD11c+APC/CD80+PE/CD86+ PE-Cy7; T helper cells were defined as CD3^+^-APC/CD4^+^-FITC; Cytotoxic T cells were defined as CD3^+^-APC / CD8^+^-PE; IFN-γ^+^producting CD8+T cells were defined as CD^3+^-APC / CD^8+^-PE / IFN-γ^+^-PE-Cy7; The memory T cells cells were defined as CD3^+^-APC / CD8^+^-PE / CD44^+^-PE-Cy7 / CD62L^+^-Percp-Cy5.5.


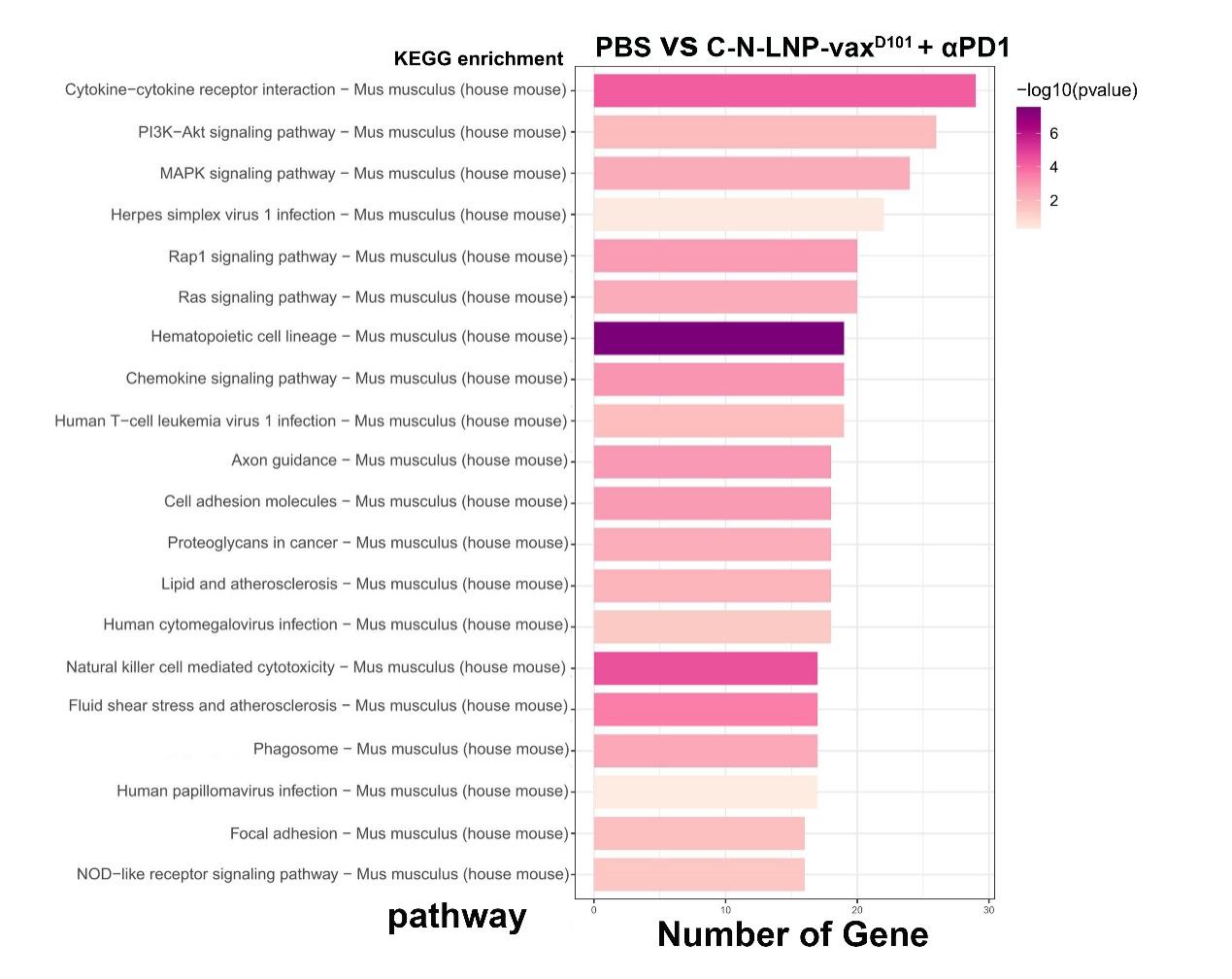


**Figure S25.** Volcano plot displaying the up-regulated, no-significantly changed, or down-regulated genes when comparing the C-N^(MC38)^-LNPs-vax^D18^ and αPD1-treated mice with PBS treated group.


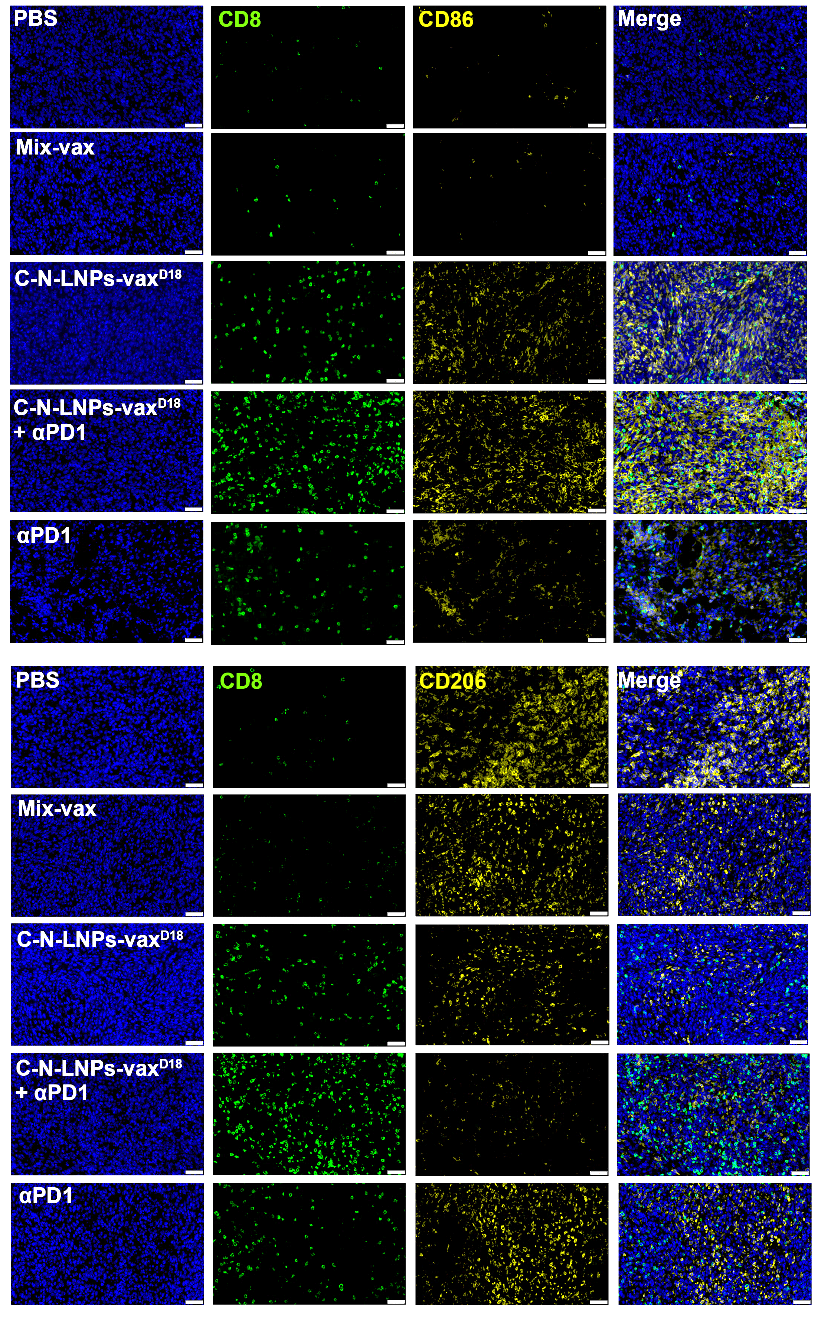


**Figure S26**. Multiplex immunoﬂuorescence staining for the CD8, CD86, and CD206 biomarkers for representing TILs, M1-TAMs, and M2-TAMs in MC38 tumors after different treatment as indicated.
